# Schisandrin B Suppresses Colon Cancer Growth by Inducing Cell Cycle Arrest and Apoptosis: Molecular Mechanism and Therapeutic Potential

**DOI:** 10.1021/acsptsci.4c00009

**Published:** 2024-02-22

**Authors:** Vanessa Anna Co, Hani El-Nezami, Yawen Liu, Bonsra Twum, Priyanka Dey, Paul A. Cox, Shalu Joseph, Roland Agbodjan-Dossou, Mehdi Sabzichi, Roger Draheim, Murphy Lam Yim Wan

**Affiliations:** † School of Biological Sciences, Faculty of Science, Kadoorie Biological Sciences Building, The University of Hong Kong, Pokfulam Hong Kong; ‡ Institute of Public Health and Clinical Nutrition, University of Eastern Finland, FI-70211 Kuopio, Finland; § School of Pharmacy and Biomedical Sciences, Faculty of Science and Health, University of Portsmouth, Portsmouth PO1 2DT, United Kingdom; ∥ Division of Microbiology, Immunology and Glycobiology, Department of Laboratory Medicine, Faculty of Medicine, Lund University, 222 42 Lund, Sweden

**Keywords:** apoptosis, cell cycle arrest, molecular docking, novel colon cancer treatment, Raman spectral change, schisandrin B

## Abstract

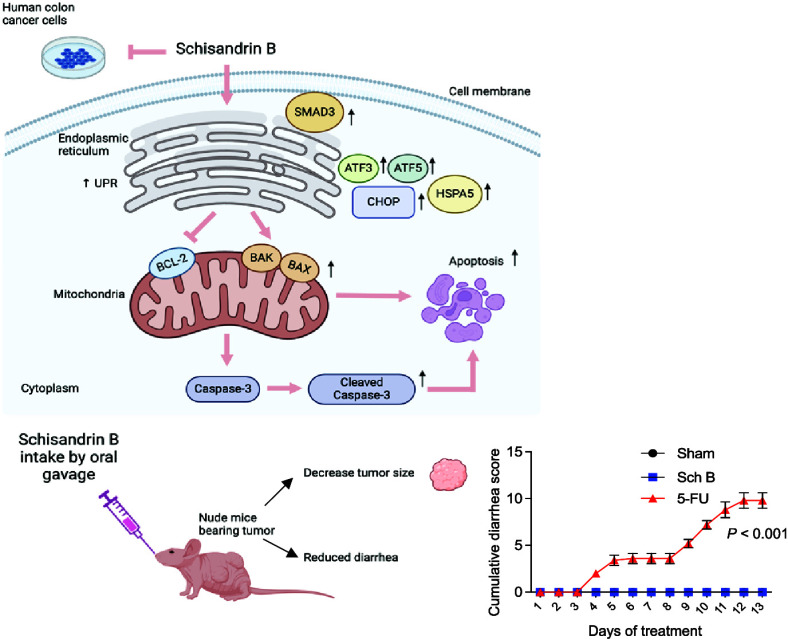

Colon cancer is among the most lethal and prevalent malignant tumors in the world, and the lack of effective therapies highlights the need for novel therapeutic approaches. Schisandrin B (Sch B), a lignan extracted from the fruit of*Schisandra chinensis*, has been reported for its anticancer properties. However, to date, no studies have been done to characterize the exact molecular mechanisms underlying the antitumorigenic effects of Sch B in colon cancer. This study aimed to explore the antitumorigenic effects of Sch B in colon cancer and to understand the underlying therapeutic mechanism. A comprehensive analysis of the molecular mechanism underlying the antitumorigenic effects of Sch B on human colon cancer cells was performed using a combination of Raman spectroscopy, RNA-seq, computational docking, and molecular biological experiments. The *in vivo* efficacy was evaluated by a mouse xenograft model. Sch B reduced cell proliferation and triggered apoptosis in human colon cancer cell lines. Raman spectroscopy, computational, RNA-seq, and molecular and cellular studies revealed that Sch B activated unfolded protein responses by interacting with CHOP and upregulating CHOP, which thereby induced apoptosis. CHOP knockdown alleviated the Sch B-induced reduction in cell viability and apoptosis. Sch B reduced colon tumor growth *in vivo*. Our findings demonstrated that Sch B induced apoptosis and inhibited cell proliferation and tumor growth *in vitro* and *in vivo*. These results provided an essential background for clinical trials examining the effects of Sch B in patients with colon cancer.

Colorectal cancer (CRC) is the third most common cancer, with over 1.9 million new cases, and the second leading cause of cancer-related death, with over 930,000 deaths.^1^ It is estimated that the global burden of CRC will increase to 3.2 million new cases and 1.6 million deaths by 2040.^2^ Several risk factors have been identified over the years, including age, family history, diet, etc. Although CRC is more prevalent in Western countries, increasing rates of CRC have been reported in countries where the risk is historically low.^2^ Current management of CRC is usually chemotherapy accompanied by surgery or radiotherapy.^3^ However, these conventional cancer therapies are often accompanied by severe side effects and significant mortality. For example, the response rate of 5-fluorouracil (5-FU) in advanced CRC is less than 15%, and long-term administration of 5-FU impairs the antitumor immune response in patients.^4^ Therefore, the identification of a novel but less toxic therapeutic strategy is urgently needed in this patient group.

In recent decades, several lines of scientific evidence suggest that polyphenols, which are found abundantly in plant-based foods and drinks, have shown significant efficacy in cancer development, including chemoprevention and anticancer ability. The former is demonstrated in multiple *in vitro*, *in vivo*, and epidemiological studies in various cancers, and it is attributed primarily to polyphenols’ potent antioxidative activities.^5−7^ The latter is achieved by pro-oxidant actions in cancer cells, which in turn induce cell cycle arrest, apoptosis, and inhibition of cancer cell proliferation.^8^ So far, there are a number of studies published exploring the effect of polyphenols on colorectal cell lines or animal models.^9−11^

Schisandrin B (Sch B), a lignan extracted from the fruit of *Schisandra chinensis*, has been reported for its anticancer properties in various cancers, for example, liver cancer,^12^ breast cancer,^13,14^ ovarian cancer,^15^ glioma,^16^ osteosarcoma,^17^ and gastric cancer.^18,19^ More recently, studies have shown that Sch B was able to prevent or treat colitis-associated colon cancer.^20,21^ Although the antitumor activities of Sch B have been confirmed in various cancer types, the potential mechanism of Sch B seems much more complex, ranging from cell cycle arrest to programmed cell death. For example, in liver cancer, Sch B induced G0/G1 cell cycle arrest and apoptosis by upregulating caspase-3 and Bcl-2 family members in cholangiocarcinoma (CCA), and reduced tumor growth in a xenograft mouse model.^12^ In breast cancer, Sch B attenuated metastasis in an animal model by modulating the epithelial-to-mesenchymal transition (EMT) or STAT3 pathway.^13,14^ In gallbladder cancer, Sch B inhibited cell proliferation and induced apoptosis by regulating apoptosis-related protein expression, such as BAX, cleaved caspase-3, cleaved caspase-9, BCL-2 *in vitro*, and suppressed tumor growth *in vivo*.^22^ In another study by Li et al., Sch B was shown to induce glioma cell apoptosis by diminishing the mitochondrial membrane potential (ΔΨm). Other studies have also reported that Sch B induces apoptosis and cell cycle arrest via Wnt/β-catenin,^23^ PI3K/AKT,^17,23^ NF-κB, and p38 MAPK signaling pathways.^24^ However, none of these studies have fully characterized the mechanism of the antitumorigenic effect of Sch B, especially in colon cancer.

Accordingly, we hypothesized that Sch B would exhibit a significant therapeutic effect against CRC as it does in other cancers. Here, we investigated the molecular mechanism for the antitumorigenic effect of Sch B on colon cancer through a combination of Raman spectroscopy, RNA-seq, computational docking, and molecular biological experiments. Our findings suggest that Sch B has therapeutic potential against colon cancer through its ability to induce apoptosis, inhibiting cell proliferation and reducing tumor growth. We propose that Sch B should be further explored as a novel and more specific approach for colon cancer treatment. If these findings are successfully translated into clinics, millions of patients with colon cancer will benefit from this approach.

## Results

### Sch B Inhibited Colon Cancer Growth in Human Colon Cancer Cells

**Figure 1:**
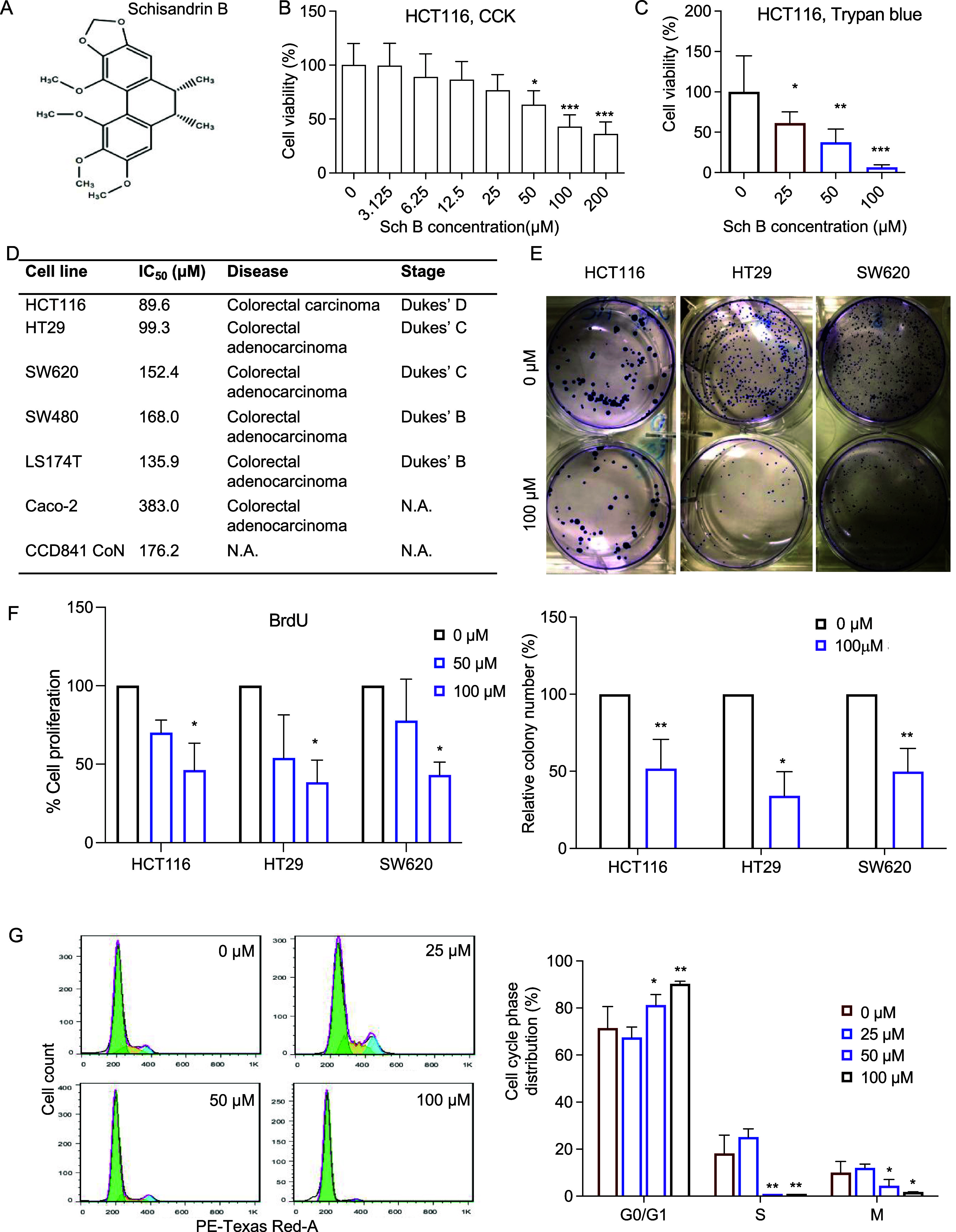
Schisandrin B inhibits the proliferation of human colon cancer cells. (A) The chemical structure of Schisandrin B (Sch B). (B, C) The viability of human HCT116 colon cancer cells was incubated with various concentrations of Sch B for 48 h, quantified by the Cell Counting Kit-8 (CCK-8) assay (B) and trypan blue exclusion assay (C) (*n* = 3 experiments). (D) Half-maximal inhibitory concentrations (IC_50_) of human colon cancer cell lines HCT116, HT29, SW480, SW620, Caco-2, and LS174T, and the normal human colon cell line CCD 841 CoN. (E) Colony assay showing the long-term effects of Sch B (100 μM) on the colony-forming potential of HCT116, HT29, and SW620 cells (*n* = 4–5 experiments). (F) Evaluation of BrdU incorporation as an index of DNA synthesis after Sch B (50 and 100 μM) treatment (*n* = 3 experiments). (G) Cell cycle analysis of HCT116 cells treated with 0, 25, 50, and 100 μM Sch B for 48 h (*n* = 6 experiments). Values are presented as mean ± SD, analyzed by the Kruskal–Wallis test with Dunn’s correction (B, F), One-way ANOVA with Holm–Šídák’s multiple comparisons (C, G) or Mann–Whitney *U*-test (E). *P < 0.05, **P < 0.01, ***P < 0.001, compared with the control (i.e., 0 μM Sch B).

The chemical structure of Sch B is depicted in Figure 1A. CCK-8 assay was performed to determine the effects and concentrations of Sch B on multiple CRC cell lines at different stages. Sch B displayed a concentration-dependent effect on all of the CRC cell lines (*P* < 0.05), except Caco-2 cells. Although Sch B also lowered the viability of the normal cell line CCD 841 CoN, the change was not statistically significant. Based on the half-maximal inhibitory concentration (IC_50_) calculated from the CCK-8 assay, HCT116, HT29, and SW620 cell lines were found to be the most sensitive to Sch B treatment and were therefore used for subsequent experiments (Figures 1B,D and S1). Trypan blue exclusion and BrdU-ELISA assays were also performed to confirm the results of the CCK assay (Figure 1C,F). In addition, the long-term antiproliferative effect of Sch B on CRC cells was assessed by a clonogenic survival assay. Sch B significantly reduced the colony-forming abilities of HCT116, HT29, and SW620 after 48 h treatment. The loss of cell viability was irreversible after 14 days (Figure 1E).

We further analyzed the cell cycle distribution of the Sch B-treated HCT116 cells. The results showed that Sch B treatment led to a decrease in the cell proportion in the S and M phases and an accumulation in the cell proportion in the G0/G1 phase (Figure 1G). These data indicate that Sch B arrests HCT116 cells in the G0/G1 phase of the cell cycle and subsequently blocks cell growth.

### Sch B Promoted Apoptosis in Human Colon Cancer Cells

**Figure 2:**
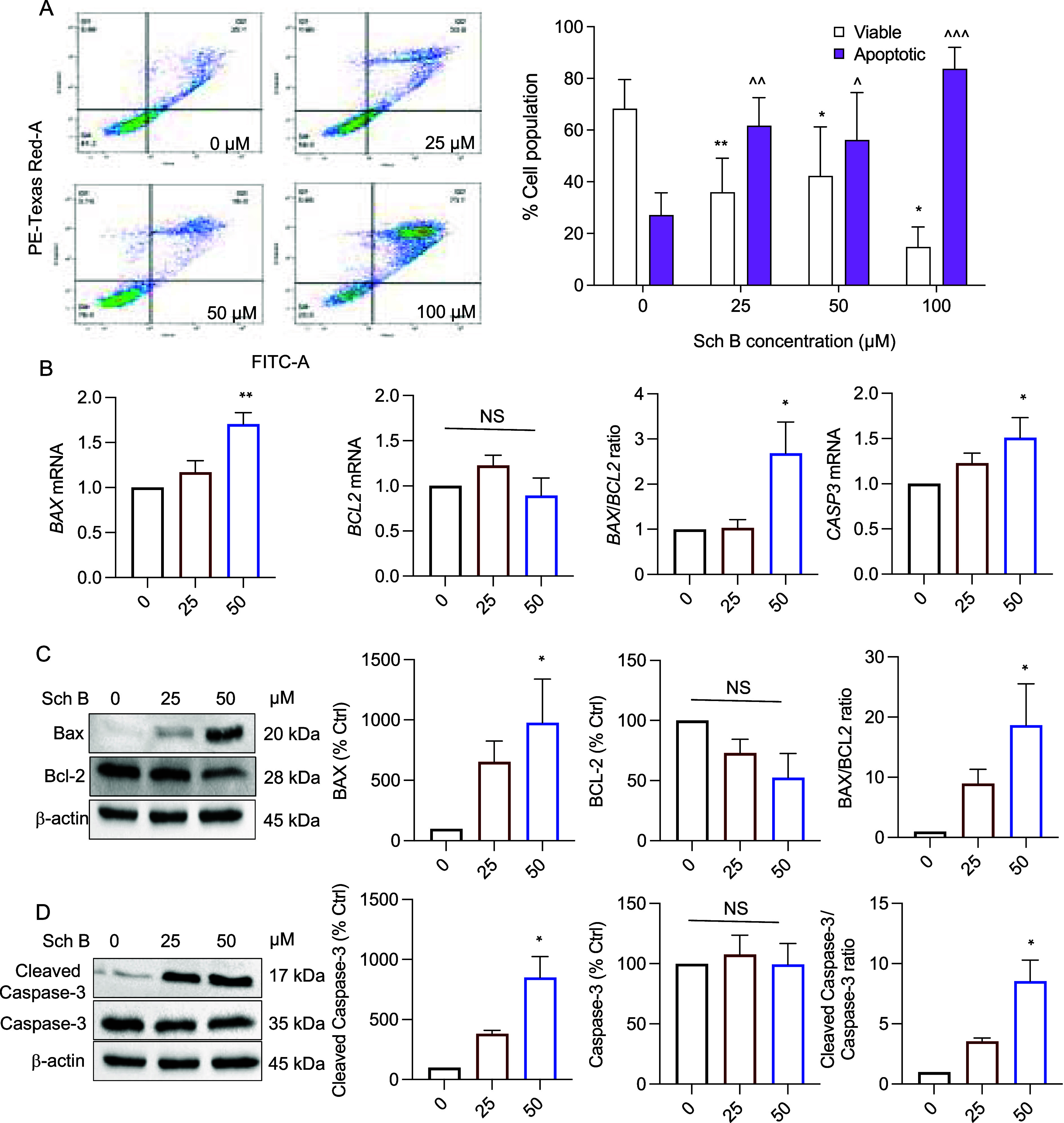
Schisandrin B promoted apoptosis in HCT116 cells. (A) Evaluation of apoptosis rates of HCT116 cells incubated with 0, 25, 50, and 100 μM Schisandrin B (Sch B) for 48 h by Annexin V/PI flow cytometry (*n* = 5 experiments). (B) mRNA expression levels of *BAX*, *BCL2*, and *CASP3* in cells treated with 0, 25, and 50 μM Sch B for 48 h. The mRNA expression levels were normalized to those of GAPDH. (C, D) Immunoblot analysis of BAX, BCL-2, and cleaved caspase-3 and caspase-3. The protein levels were normalized to β-actin levels (*n* = 3 experiments). Values are presented as mean ± SD, analyzed by one-way ANOVA with Holms–Šídák’s multiple comparisons (A) or Kruskal–Wallis test with Dunn’s correction (B-D). *P < 0.05, **P < 0.01, ***P < 0.001, compared with the control (i.e., 0 μM Sch B). NS = no significance.

Cell apoptosis was analyzed by using Annexin V/PI staining. The flow cytometry results showed an accumulation of apoptotic and necrotic cells in the human colon cancer cells exposed to increasing concentrations of Sch B (Figure 2A).

qPCR and Western blotting were also used to analyze the mRNA and protein levels of several important apoptosis-related markers (*BAX*, *BCL2*, and *CASP3*) (Figure 2B–D). The results of qPCR showed that the mRNA levels of apoptotic genes *BAX* and *CASP3* were elevated in the Sch B-treated cells, but no change in the antiapoptotic gene *BCL2* mRNA was observed. Since the balance between pro-apoptotic and antiapoptotic proteins of the BCL family plays a key role in the regulation of the intrinsic pathway of cell apoptosis, we calculated the BAX/BCL-2 ratio as a proper indicator of cell apoptosis. Sch B significantly increased the the BAX/BCL-2 ratio in a concentration-dependent manner (Figure 2B). Similarly, Sch B treatment significantly increased the BAX and cleaved (active) caspase-3 protein levels, while there was no difference in the pro-caspase-3 protein levels among all treatment groups. The increase in BAX/BCL-2 and active caspase-3/pro-caspase-3 ratios indicated that Sch B might induce human colon cancer cell death by promoting apoptosis (Figure 2C,D).

### Raman Spectroscopy Revealed Changes in Molecular Signatures in Cells after Sch B Treatment

**Figure 3:**
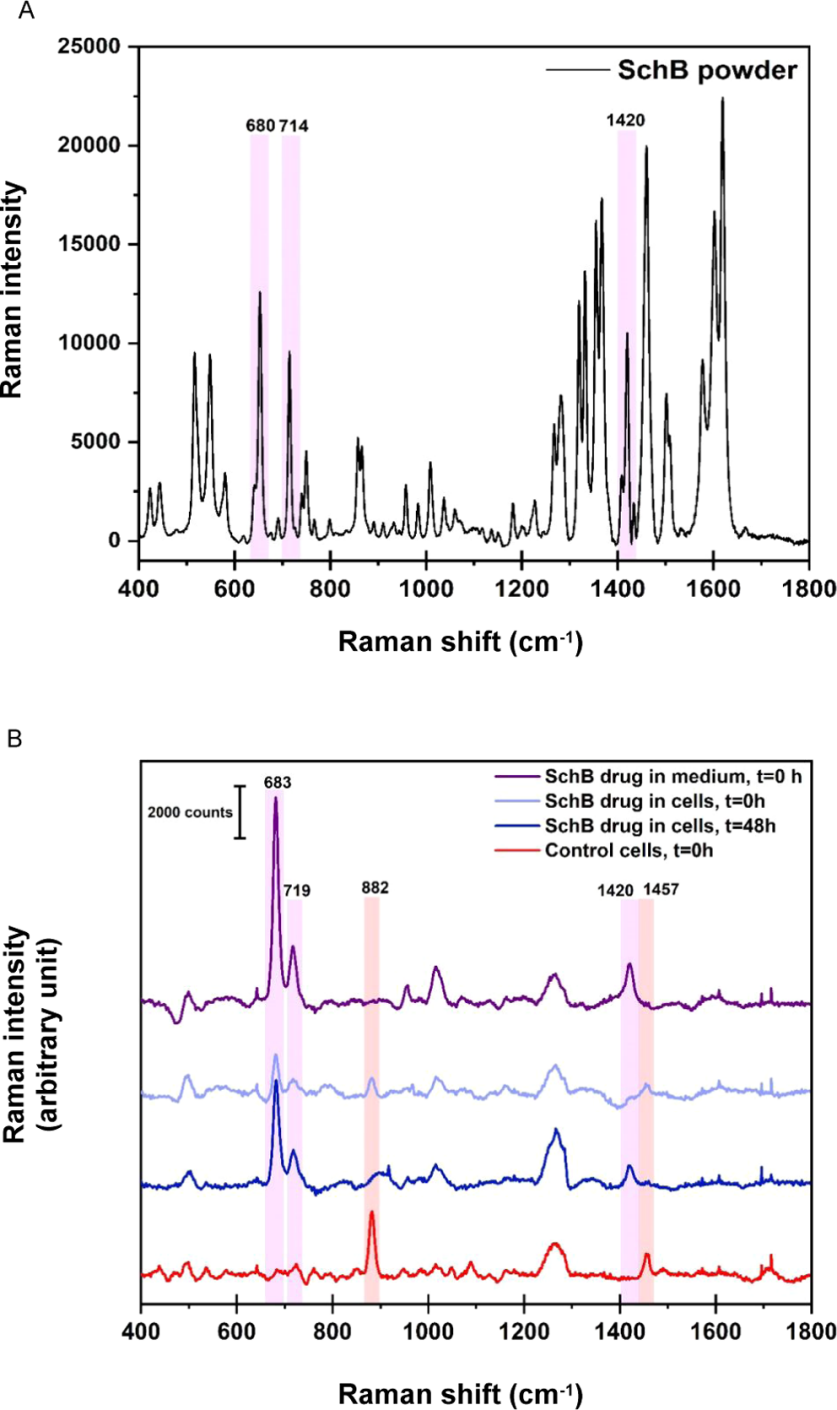
Raman spectroscopy analysis of the uptake of Schisandrin B and the effect on human colon cancer cells. (A) The Raman spectrum of Schisandrin B (Sch B) powder samples with several signature peaks at 680, 714, and 1420 cm^–1^ (depicted with pink bars). (B) The spectra of cell lysates and cell-free supernatants from cells cultured in the presence or absence of Sch B. Cell lysates and cell-free supernatants were obtained from human colon cancer cells exposed to 50 μM Sch B (i.e., 20 μg/mL) and analyzed by an in-house compact Raman spectrometer. The degree of Sch B uptake by the cells after 0 and 48 h was quantified from the relative intensities of the Raman peaks of Sch B (683, 719, and 1420 cm^–1^).

To uncover the underlying mechanism for the anticancer effects of Sch B, we first explored the uptake of Sch B and its effect on human colon cancer cells by using nondestructive, label-free Raman spectroscopy. The Raman spectrum of the Sch B powder sample was analyzed by a commercial Renishaw Raman spectrometer with several signature peaks occurring at 680, 714, and 1420 cm^–1^ (depicted with pink bars) (Figure 3A). Cell lysates and cell-free supernatants were obtained from human colon cancer cells exposed to 50 μM Sch B (i.e., 20 μg/mL) and analyzed by an in-house compact Raman spectrometer. The spectra of cell lysates and cell-free supernatants from cells cultured in the presence or absence of Sch B are shown in Figure 3B. A slight Raman shift was detected in cell lysates and cell-free supernatants containing Sch B compared to Sch B powder, where the signature Raman peaks occurred at 683, 719, and 1420 cm^–1^ (depicted with pink bars). Such a shift in the peak positions may be due to the differences in the spectral resolution of the two Raman systems and/or solvation caused by Sch B in the cell media. However, these Raman peak positions were absent in the cell lysate from cells without Sch B treatment, confirming that these peaks were related to Sch B.

A visual comparison of the spectral intensities further showed differences in the Raman spectra of the cell lysates at 0 and 48 h time points (Figure 3B). The peaks pertaining to the Sch B and cellular components are depicted by pink and red bars, respectively. It is interesting to note that Raman peaks were observed at 882 and 1457 cm^–1^ in the spectrum of cell lysates obtained at 0 h but not at 48 h, which are often associated with C–C skeletal vibrations, CH_2_ scissoring and CH_3_ deformation vibrations, suggesting the presence of aliphatic amino acids and/or glucose, respectively. The identified peaks may be considered as potential indicators of the drug’s effect on the metabolism or death of the cells, and further experiments are required to verify this.

Furthermore, cellular uptake is another important contributor to the efficiency and biological activity of novel drugs. To monitor Sch B uptake by the cells, relative intensities of the Raman peaks of Sch B (683, 719, and 1420 cm^–1^) were measured after 0 and 48 h of Sch B treatment. At the 0 h time point, rapid uptake (17%) of Sch B into the cells occurred. At 48 h, the level of Sch B uptake by the cells was significantly increased to about 42%, which corresponds to approximately 12.6 μg of Sch B taken up by the cells (initial cell number: 2.16 × 10^6^, 5.83 pg Sch B per cell).

### Sch B Induced Differential Gene Expression in Cancer Cells

**Figure 4:**
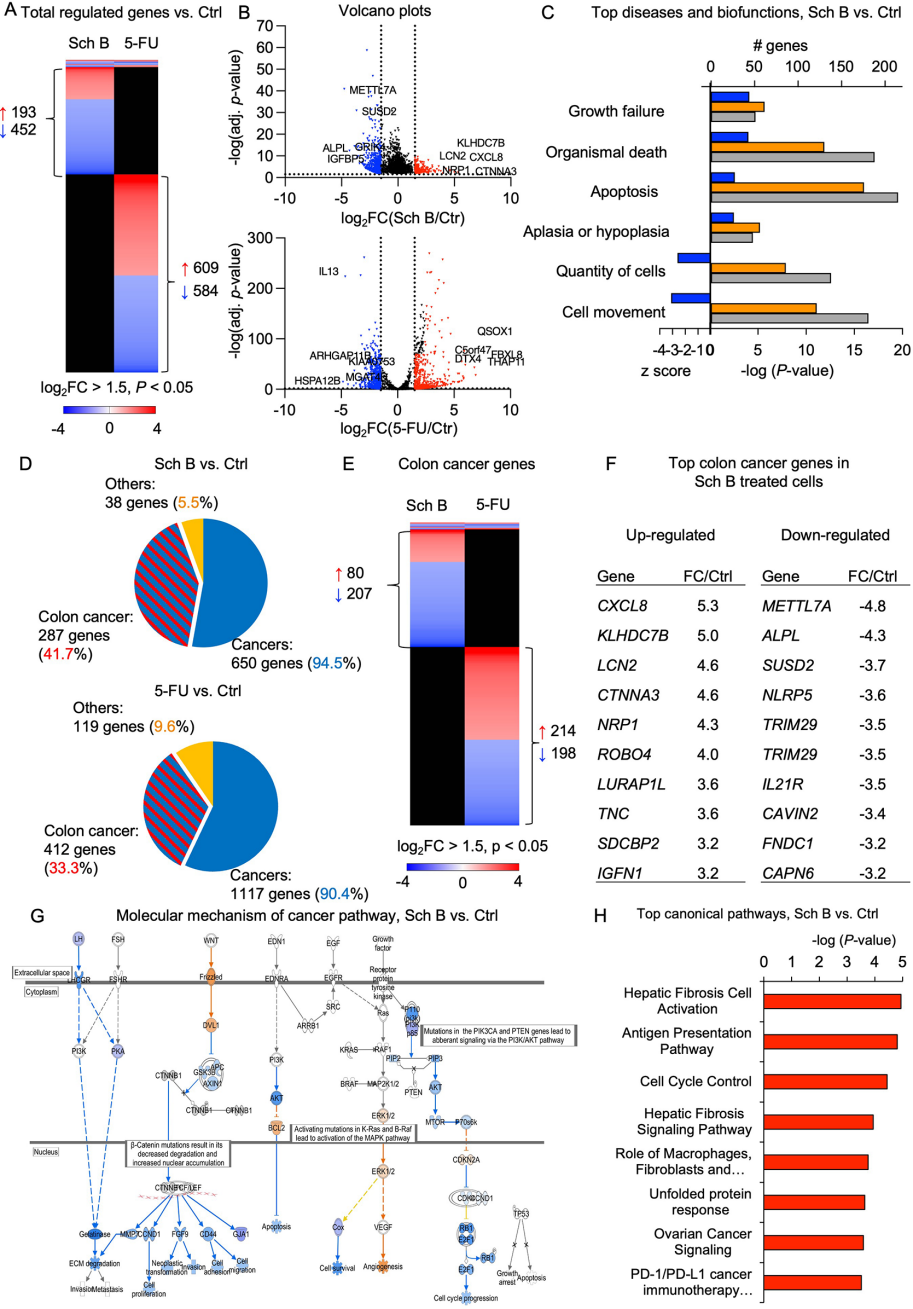
RNA-sequencing analysis of HCT116 cells treated with Schisandrin B or 5-fluorouracil. (A) Heatmap comparing gene expression profiles of cells after exposure to 50 μM Schisandrin B (Sch B) or 100 μM 5-fluorouracil (5-FU) compared to untreated control (red, upregulated; blue, downregulated; black, not regulated; cutoff log_2_FC ≥ 1.5, *P* < 0.05, *n* = 3 samples per group). (B) Volcano plots showing differentially expressed genes in Sch B- or 5-FU-treated cells. (C) Biofunctional analysis showed that Sch B affected genes related to cell growth, cell movement, organism death, and apoptosis (blue, *Z*-score; orange, *P*-value; gray, no. of genes). (D) Pie chart of the genes regulated in response to Sch B or 5-FU treatment. Gene categories were identified by biofunction analysis. (E) Heatmap comparing colon cancer-related gene expression profiles of cells after exposure to 50 μM Sch B or 100 μM 5-FU compared to the untreated control. (F) Top colon cancer-related genes in cells treated with Sch B. (G) IPA analysis showing the inhibition of the cancer pathway in Sch B-treated cells (orange, upregulated genes; blue, downregulated genes). (H) Top canonical pathway affected by Sch B.

In order to identify genome-wide alterations in the transcriptome of human colon cancer cells treated with Sch B, total RNA was extracted from cells and subjected to RNA-seq. Gene expression profiles were compared between untreated cells and cells after exposure to Sch B. 5-FU-treated cells served as positive controls (Figure 4).

Our RNA-seq results revealed the major differences between Sch B- and 5-FU-treated cells compared to the untreated cells. There were 193 upregulated genes and 452 downregulated genes in the Sch B group (cutoff log_2_FC ≥ 1.5, *P* < 0.05) (Figure 4A). The top-regulated genes included *METTL7A*, *ALPL*, *SUSD2*, *IGFBP5*, *CORO1A*, *CXCL8*, *LCN2*, *NRP1*, and *CTNNA2*, which are involved in tumor proliferation, angiogenesis, migration, metastasis, and tumor immunity. In 5-FU-treated cells, 609 genes were upregulated, and 584 genes were downregulated. The top-regulated genes included *KIAA0753*, *IL13*, *HSPA12B*, *MGAT4B*, *THAP11*, *QSOX1*, *DTX4*, and *FBXL8*, which are related to cell movement, cancer invasion and metastasis, drug resistance, inflammation, and immune overactivation (Figure 4B). Functional analysis revealed that Sch B affected genes related to cell growth, cell movement, organism death, and apoptosis, which was consistent with the results from previous cell death and functional assays (Figure 4C).

While only 43 genes were commonly regulated between Sch B- and 5-FU-treated cells, indicating that Sch B and 5-FU had very different targets for gene regulation, Sch B treatment was also as efficient as 5-FU, as defined by strong effects on cancer gene networks (Figure 4D–F). 95% of all regulated genes in the RNA extracts from cells treated with Sch B were cancer-related. This included a subset of colon cancer-related genes that were inhibited (41.7%) (Figure 4D–F).

The molecular mechanisms of cancer pathway genes were deactivated, including *COX*, *GJA1*, *CD44*, *FGF9*, and *CCND1* (Figure 4G). Further pathway analysis of the RNA-seq data also detected several major pathways regulated by Sch B, including hepatic fibrous and ovarian cancer signaling, cell cycle control, unfolded protein response, and PD-1/PD-L1 cancer immunotherapy (Figure 4H).

### Sch B Promoted ER Stress-Dependent Apoptosis in Colon Cancer Cells via Activation of CHOP Signaling

**Figure 5:**
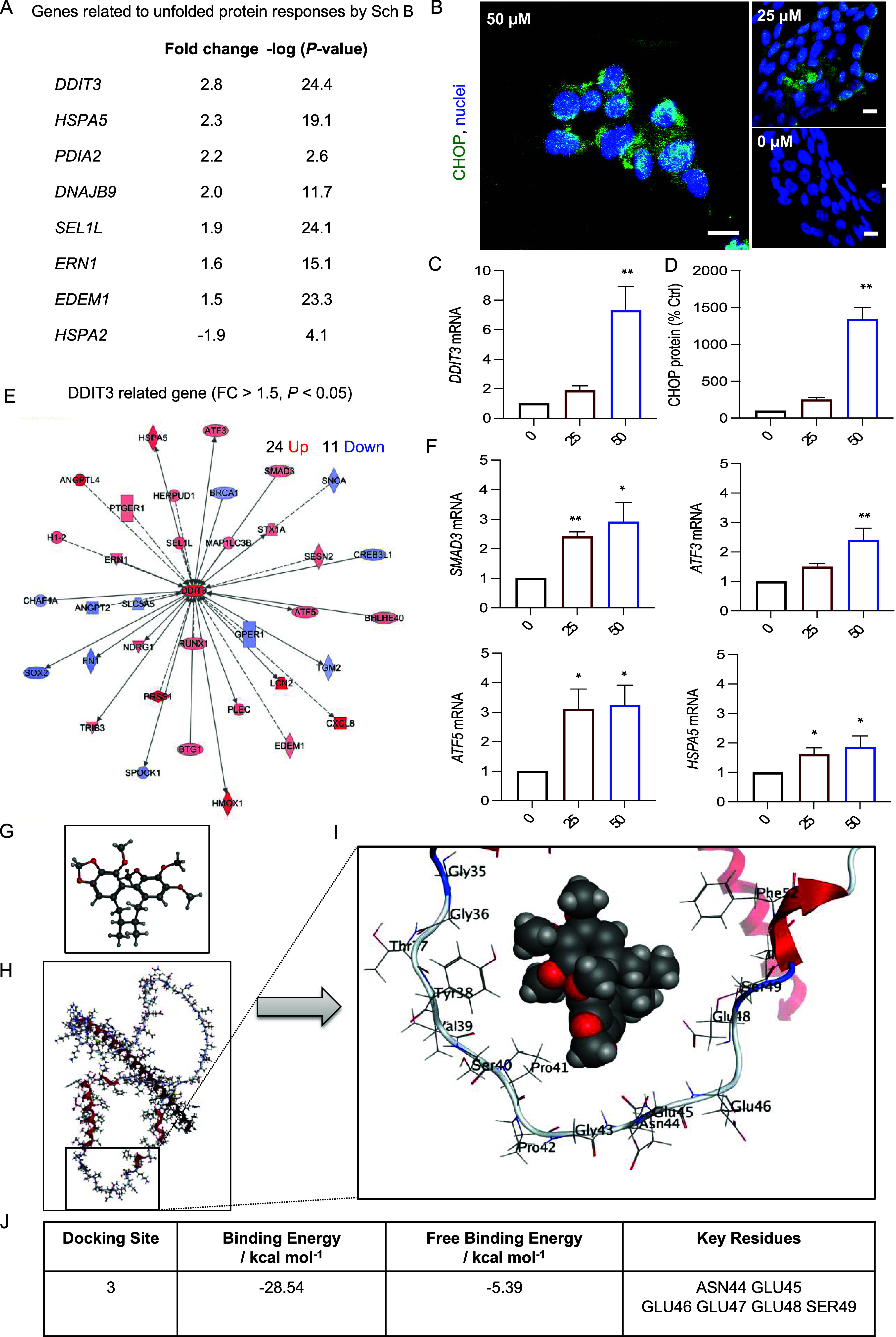
Schisandrin B activated the CHOP signaling pathway of the unfolded protein response. (A) Ingenuity pathway analysis (IPA) identified *DDIT3* as one of the top upregulated genes. (B) Confocal imaging of the CHOP protein (green, CHOP; blue, nuclei; scale bars, 10 μm) (*n* = 3 experiments). (C) mRNA levels of DDIT3 by qPCR (*n* = 4 experiments). (D) Protein expression levels of CHOP by immunofluorescence staining (right). For confocal image quantification, 50 cells were counted per sample (*n* = 3 experiments). (E) *DDIT3* and DDIT3-dependent gene network (red, upregulated genes; blue, downregulated genes; cutoff log_2_FC ≥ 1.5, *P* < 0.05). (F) mRNA expression of DDIT3-dependent gene expression quantified by qPCR (*n* = 4 experiments). Values are presented as mean ± SD, analyzed by the Kruskal–Wallis test with Dunn’s correction (C, D, F). *P < 0.05, **P < 0.01, ***P < 0.001, compared with the control (i.e., 0 μM Sch B). (G–J) The proposed molecular model of Sch B binding to CHOP. (G) The 3D chemical structure of Sch B. (H) The CHOP protein structure. (I) The energy-minimized location of the Sch B molecule docked with CHOP protein. The rectangle in (H) shows a part of the CHOP structure represented in (I). (J) The docking site with the lowest binding energy and key residues is involved in the interaction.

Unfolded protein response (UPR) is a known cellular defense mechanism in response to endoplasmic reticulum (ER) stress and is implicated in cancer progression and pathogenesis. Our results showed that unfolded protein responses are one of the top canonical pathways regulated by Sch B. Analysis of genes involved in the unfolded protein response pathway revealed DDIT3 as the top-regulated gene that was upregulated by Sch B treatment (Figure 5A).

To confirm the results of RNA-seq, we performed real-time qPCR and immunofluorescence staining for DDIT3 (CHOP) expression (Figure 5B,C). Consistent with the RNA-seq data, both mRNA and protein levels of DDIT3 (CHOP) were remarkably upregulated in cells following Sch B treatment (Figure 5C,D). Further transcriptomic analysis identified a network of DDIT3 (CHOP)-dependent genes being upregulated (Figure 5E). qPCR analysis confirmed that the mRNA expression of several CHOP-dependent genes, including *SMAD3*, *ATF3*, *ATF5*, and *HSPA5*, was significantly upregulated in Sch B-treated cells (Figure 5F).

To investigate the interaction between the CHOP protein and the Sch B molecule, molecular docking was performed (Figure 5G–J). Nine potential binding sites were identified using the Site Finder tool within the Molecular Operating Environment (MOE) program.^25^ Of these sites, two had significantly higher binding energies than the other seven sites (Table S1). These two sites (sites 3 and 7) are very close to each other in the structure since they share common residues (GLU46, GLU47, and SER49). The energy-minimized location of the Sch B molecule at site 3 is shown in Figure 5I,J.

**Figure 6:**
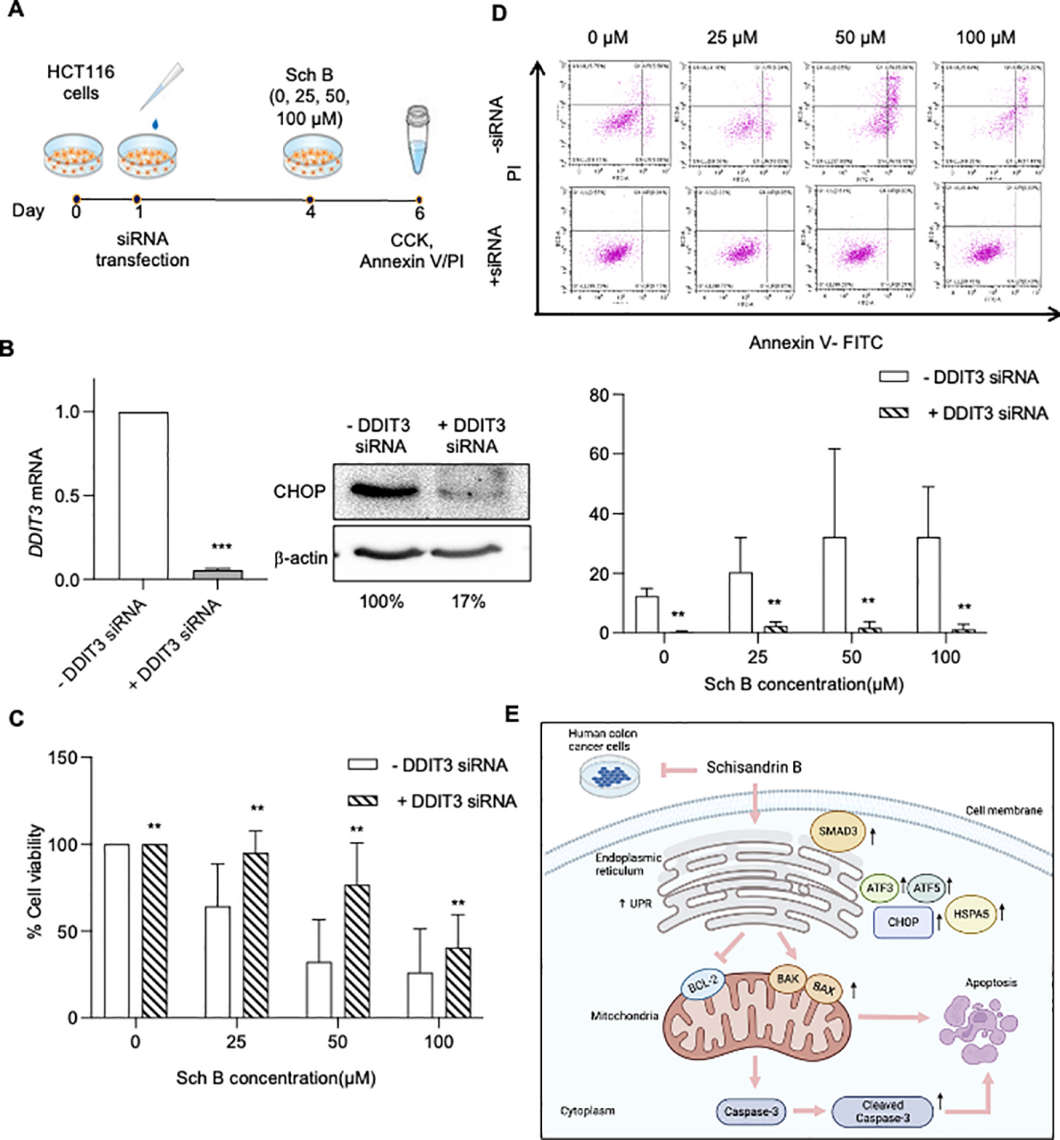
Schisandrin B promoted CHOP-dependent apoptosis in human colon cancer cells. (A) Schematic of the protocol used to treat the HCT116 cells. Cells were treated with siRNA for 72 h and then treated with or without 0, 25, and 50 μM Schisandrin B (Sch B) for 48 h. Cells were harvested for the CCK assay and Annexin V/PI flow cytometry. (B) mRNA and protein expression levels of DDIT3/CHOP in siCHOP-transfected cells. The mRNA and protein expression levels were normalized to those of GAPDH and β-actin, respectively (*n* = 2 experiments). (C) The viability of siCHOP-transfected cells incubated with 0, 25, 50, and 100 μM Sch B for 48 h, as quantified by the CCK assay (*n* = 3 experiments). (D) Evaluation of apoptosis rates of siCHOP-transfected HCT116 cells by Annexin V/PI flow cytometry (*n* = 3 experiments). (E) Schematic of CHOP-dependent induction of apoptosis by Sch B. Values are presented as mean ± SD, analyzed by Wilcoxon-matched-pair signed-rank test (B), One-way ANOVA with Holm–Šídák’s multiple comparisons (C, D). *P < 0.05, **P < 0.01, and ***P < 0.001, compared with the control (i.e., 0 μM Sch B).

Furthermore, it is well documented that activation of CHOP during ER stress can induce cell cycle arrest and oxidative stress, leading to cell death and apoptosis.^26^ To determine the role of ER stress in Sch B-induced growth inhibition, we performed siRNA-mediated silencing of CHOP in HCT116 cells (Figure 6A,B). Using the CCK-8 assay and Annexin V/PI staining, we found that CHOP knockdown moderately restored the Sch B-induced changes in cell viability, and Sch B-induced apoptosis was attenuated (Figure 6C,D). These results suggest that CHOP activation is involved in the regulation of Sch B-triggered responses (Figure 6E).

### Sch B Suppressed Colon Cancer Growth In Vivo

**Figure 7:**
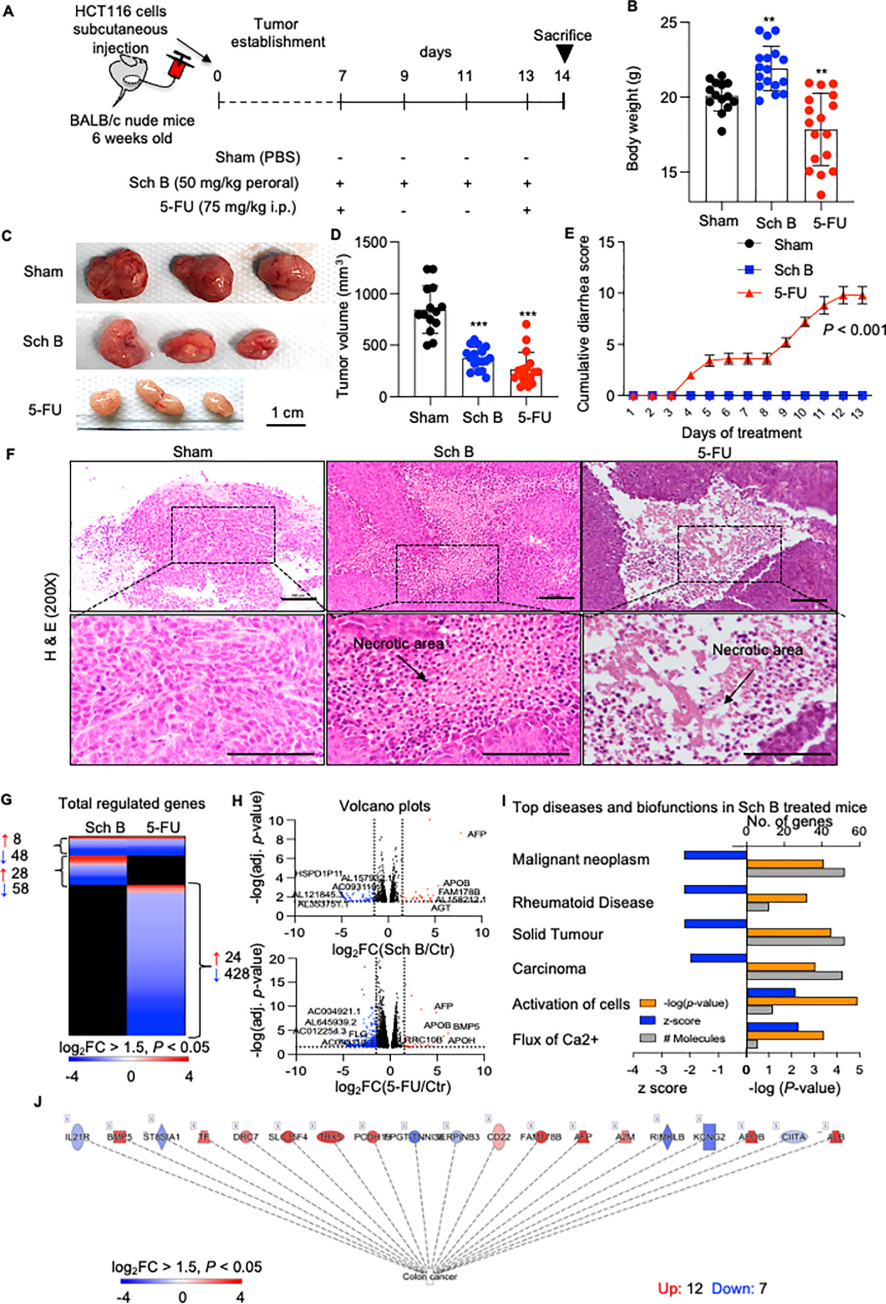
Therapeutic efficacy of Schisandrin B in a mouse xenograft model of human colon cancer. (A) Schematic of the protocol used to treat HCT116 colon cancer xenografts in nude mice. Six-week-old male BALB/C nude mice were subcutaneously injected with HCT116 cells. One week after tumor injection, the mice were given either perorally Schisandrin B (Sch B) (50 mg/kg) every other day or injected intraperitoneally once a week with 5-fluorouracil (5-FU) (75 mg/kg). The treatment lasted for 14 days. (B) Body weights of mice in different treatment groups. (C) Representative images of tumors from each treatment group. Scale bars, 1 cm. (D) Tumor volume. (E) Mice were monitored daily after treatment to observe diarrhea. A diarrhea assessment score was given to each mouse based on the wetness and hardness of the stool. 0 = normal (normal stool or absent); 1 = slight (slightly wet and soft stool); 2 = moderate (wet and unformed stool with moderate perianal staining of the coat); and 3 = severe (watery stool with severe perianal staining of the coat). (F) Microscopic examination of hematoxylin and eosin (H&E) stained tumor sections (200×). Representative sections; scale bars, 100 μm. Arrows indicate the areas of necrosis. (G) Heatmap comparing gene expression profiles of tumors from Sch B- and 5-FU-treated mice compared to untreated controls (red, upregulated; blue, downregulated; black, not regulated; cutoff log_2_FC ≥ 1.5, *P* < 0.05, *n* = 3 mice per group). (H) Volcano plots showing differentially expressed genes in tumors from Sch B- or 5-FU-treated mice. (I) Top diseases and biofunctions of genes significantly regulated by Sch B (blue, *Z*-score; orange, *P*-value; gray, no. of genes). (J) Several colon cancer genes were significantly regulated in tumors obtained from mice receiving Sch B compared to those in the untreated control (red, upregulated genes; blue, downregulated genes).

To characterize the *in vivo* antitumor effect of Sch B, we generated nude mice bearing HCT116 cell xenografts. The mice were treated perorally every other day with Sch B at a dose of 50 mg/kg body weight for 1 week. 5-Fluorouracil (5-FU)-injected mice served as positive controls (Figure 7A). The results showed that the tumor volume was significantly reduced in Sch B-treated mice compared to that in sham-treated mice. In addition, the tumor weight was reduced in the Sch B-treated mice, while the body weight remained unchanged between the Sch B- and sham-treated groups (Figure 7B–D). Since diarrhea is one of the side effects reported in 5-FU treatment, the animals were monitored daily for diarrhea from the start of the treatment. The sham- and Sch B-treated mice did not show any incidence of diarrhea. The animals in the 5-FU group had a significantly higher cumulative diarrhea assessment score than the other groups from day 4 (Figure 7E). Histopathological examinations were further performed by staining with hematoxylin and eosin (H&E) to examine any morphological changes relevant to apoptosis/necrosis in tumor tissues. The results indicated a lower tumor cell density in the Sch B and 5-FU groups than in the sham group. Tumor necrosis was observed in both Sch B- and 5-FU-treated tumor tissues, and inflammatory cell infiltration was also observed in Sch B-treated tumor tissues (Figure 7F). The prognostic and proliferation marker Ki-67 was also examined in the tumor sections from the Sch B- and 5-FU-treated mice. Sch B did not cause any significant changes in Ki-67 expression compared to 5-FU, however (Figure S2).

To characterize the tumor response to Sch B, total RNA derived from tumor tissues was subjected to RNA-seq. Gene expression profiles were compared between the sham-treated group and mice receiving Sch B. Tumor tissues from 5-FU-treated mice served as positive controls (Figure 7G–J).

Similar to our *in vitro* studies, the RNA-seq results revealed a profound difference in the gene expression profiles between the Sch B and 5-FU treatment groups compared to the sham group. There were 36 upregulated genes and 106 downregulated genes in the Sch B group (cutoff log_2_FC ≥ 1.5, *P* < 0.05) (Figure 7G). The top-regulated genes included long noncoding RNAs (lncRNAs; *Al157932*.1, *Ac093110*.1, *Al353751*.1), *Hspd111*, *Afp*, *Apob*, *Fam178b*, and *Agt*. In the 5-FU treatment group, 32 genes were upregulated, and 476 genes were downregulated. The top-regulated genes included *Bmp5*, *Apob*, *Afp*, *Apoh*, *Lrrc10b*, and *Flg*, as well as some lncRNAs (*Ac012254*.3, *Al645939*.2, *Ac004921*.1) (Figure 6H). Despite the relatively low number of genes regulated by Sch B, cancer biofunctions were markedly attenuated (*Z*-score > −2) in Sch B-treated mice compared to those in the sham group (Figure 7I). A subset of colon cancer genes was specifically regulated (Figure 7J). The biofunctional analysis further predicted a number of genes responsible for the inhibition of cell proliferation and activation of apoptosis in tumor tissues from Sch B-treated mice. Some of these genes might be worth exploring and developing as potential diagnostic or therapeutic biomarkers for colon cancer in the future (Figure S3).

Lastly, pathway analysis revealed several major pathways regulated by Sch B, including LXR/RXR activation, acute-phase response signaling, clathrin-mediated endocytosis signaling, and the production of NOx and ROS in macrophages. However, unfolded protein response signaling was not significantly affected in Sch B-treated tumor tissues (Figure S4).

## Discussion

Conventional cancer therapies are often accompanied by various side effects and organ toxicities, resulting in a lower quality of life for patients.^27^ While chemotherapy remains the mainstay of cancer treatment, it often incurs high costs.^28^ The monthly cost of 5-FU-based chemotherapy is estimated to be USD 2370 to USD 4665 for each patient, depending on the administration method.^29^ As such, there is an urgent need to develop less costly alternatives to chemotherapy for CRC that could lead to improved clinical outcomes.

Our data have discovered an entirely new approach that offers a safe and cost-effective alternative to human colon cancer treatment. Sch B, a lignan found in the fruit of*S. chinensis* (also known as five-flavor berry), offers astonishing properties and goes beyond current cancer-specific therapies in the following aspects: (1) it is a natural polyphenol with high tumor-killing capacity and a high degree of specificity; (2) it treats different stages of colon cancers, especially more effective for the late stage of colon cancers; and (3) it has shown very low toxicity against normal cells compared to currently available drugs.

Furthermore, this study is the first to provide a comprehensive understanding of the molecular mechanism underlying the antitumorigenic effect of Sch B on colon cancer through a combination of Raman spectroscopy, RNA-seq, computational docking, and molecular biological experiments. Mechanistic investigation revealed that Sch B treatment triggered apoptosis in the colon cancer cells through the activation of the ER stress pathway. Our study showed that Sch B upregulated UPR markers, such as DDIT3 and HSPA78, indicating that Sch B induces ER stress in CRC cells. ER stress is regularly reported in cancer cells.^30^ In response to ER stress, URP restores homeostasis of the ER folding environment. It can either promote cell survival or cell death in tumor cells, depending on the cell status.^31^ In cells with prolonged ER stress, C/EBP homologous protein (CHOP, also known as DDIT3) is upregulated and subsequently induces apoptosis in cancer cells via multiple signaling pathways.^32^ Previous studies have demonstrated the pro-apoptotic and antiproliferative effects of activated CHOP signaling in cancer cells.^33,34^ In mitochondria-dependent apoptosis, CHOP regulates the expression of BCL-2 family proteins and TRB3 protein.^35^ Overexpression of CHOP can induce translocation of BAX protein from the cytosol to the mitochondria, resulting in cell death.^36^ Consistent with our findings, we demonstrate that Sch B could effectively bind to the active sites of CHOP. It is interesting to note that Sch B is located away from the two functional domains in the CHOP protein, the N-terminal transcriptional activation domain and the C-terminal basic-leucine zipper domain. This would seem to be consistent with the Sch B molecule, which potentially activates the protein rather than inhibiting it. Furthermore, we showed that in cells where CHOP was silenced, the efficacy of Sch B against CRC cells was significantly reduced compared to that in nontransfected cells. These data suggest that Sch B may induce cell death through the activation of CHOP.

The therapeutic efficacy of Sch B in colon cancer was also confirmed in a mouse xenograft model in this study. While the unfolded protein response was not significantly regulated by Sch B in the mouse model, it could be due to differences in dosage and administration method as well as the larger variation among animal-derived samples. In the cell culture model, CRC cells were directly exposed to Sch B, but in the mouse model, Sch B was administered by oral gavage. Therefore, we need to take into account the bioavailability of Sch B in CRC cells compared to that in the tumor site and the potential metabolism of Sch B by the host and also gut microbiota, all of which could affect how Sch B interacts with the tumor cells. However, the studies on bioavailability and metabolism of Sch B are currently in progress in our laboratory and are thus not discussed further here.

In addition, the discovery of the role of the unfolded protein response in cancer progression is relatively new, and its mechanisms are still not fully understood.^37^ There are multiple pathways by which CHOP induces apoptosis. So far, the mitochondria-dependent pathway, PERK-ATF4-CHOP pathway, and CHOP-induced ROS have been identified.^34^ It is possible that Sch B-induced CHOP inhibits tumorigenesis via different CHOP-dependent pathways in *in vitro* and *in vivo* models. Therefore, it might be worth investigating the interaction between Sch B and CHOP signaling by examining the downstream regulators of CHOP signaling in the CRC cells and transplanted tumor tissues, respectively.

Overall, we demonstrated that Sch B induced apoptosis and inhibited cell proliferation and tumor growth *in vitro* and *in vivo*. These promising anticancer results indicate a potential and effective therapeutic strategy that uses Sch B for CRC treatment. The combination of Raman spectroscopy, RNA-seq, computational docking, and molecular biological experiments provides comprehensive information about the mechanism of the anticolon cancer effects of Sch B, making it possible to identify and develop novel molecular and biological targets for future clinical applications.

## Materials and Methods

### Chemicals and Reagents

Dulbecco’s modified Eagle’s medium (DMEM), fetal bovine serum (FBS), trypsin-EDTA (0.05%), and other cell culture reagents were purchased from Gibco-Life Technology (Eggenstein, Germany). Sch B was purchased from MedChem Express (Monmouth Junction, NJ), and 5-fluorouracil (5-FU) and crystal violet were obtained from Sigma-Aldrich (St. Louis, MO). They were dissolved in dimethyl sulfoxide (DMSO; Sigma) and stored at −20 °C until use. In addition, phosphate-buffered saline (PBS), sodium bicarbonate, diethylpyrocarbonate (DEPC), and chloroform were purchased from Sigma. Ammonium persulfate, *N*,*N*,*N*′,*N*′-tetramethylethane-1,2-diamine (TEMED), acrylamide, resolving gel buffer, stacking gel buffer, 10% (w/v) Tween 20, and 20% (v/v) sodium dodecyl sulfate (SDS) were purchased from Bio-Rad (Richmond, CA). Absolute ethanol and isopropanol were obtained from Merck (Darmstadt, Germany). RNAiso Plus, PrimeScript RT reagent kit with gDNA Eraser, and TB Green Premix Ex Taq were purchased from Takara (Otsu, Japan). Molecular Probes Dead Cell Apoptosis Kits with Annexin V for Flow Cytometry, propidium iodide, GeneJET RNA Purification kit, and Histomount were from Thermo Fisher Scientific (Dreieich, Germany). HiScript RT SuperMix for qPCR and AceQ qPCR SYBR Green Master Mix were obtained from Vazyme Biotech Co. (Piscataway, NJ).

### Cell Culture

Human colorectal adenocarcinoma HCT116, HT29, SW480, SW620, Caco-2, and LS174T, and normal human colon CCD 841 CoN cells were cultured in Dulbecco′s modified Eagle′s medium (DMEM), supplemented with 10% fetal bovine serum (FBS) (v/v) and maintained at 37 °C under a humidified atmosphere, with 5% CO_2_. Cells within 10 passages were used for all assays. All cells were screened for mycoplasma contamination with a MycoAlert mycoplasma detection kit (Lonza, Basel, Switzerland) prior to use.

### Cell Viability Assay (CCK-8 Assay)

Cell Counting Kit-8 (CCK-8) assay (Dojindo, Kunamoto, Japan) was used to assess cell viability after exposure to Sch B for the indicated time points. 10^4^ cells/well were seeded into 96-well culture plates (Corning, NY) and allowed to adhere for 24 h. Then, cells were treated with different concentrations of Sch B (0–200 μM) for 48 h. 10 μL of CCK-8 was added to the wells after treatment and incubated at 37 °C for 1 h. The absorbance of the wells was measured at 450 nm. The viability of human colon cancer cells after treatment was expressed as the proportion of optical density (OD) compared with that of the control (untreated cells). Based on the cell viability assay results, HCT116 cells were found to be the most sensitive to Sch B treatment and were, therefore, selected for use in other assays.

### Cell Cycle Flow Cytometry

Cells were seeded into 60 mm culture dishes and treated with Sch B for 48 h. The cells were washed with PBS and harvested by using trypsin-EDTA (0.05%). DMEM containing 10% FBS was used to inactivate trypsin. The cells were centrifuged at 1000 rpm for 5 min to remove the supernatant and washed twice with PBS. Then, the cells were fixed in ice-cold 70% ethanol and stored at −20 °C. Fixed cells were centrifuged at 500*g* for 5 min at room temperature and washed with PBS. The supernatant was aspirated, and cells were stained with 500 μL of propidium iodide (PI) staining solution for 30 min. A BD Bioscience FacsAria III flow cytometer (San Jose, CA) was used to analyze the fluorescence output at the FITC and PI emission wavelengths. FlowJo 7.6 software (BD Bioscience, San Jose, CA) was used for data analysis.

### Annexin V/PI Flow Cytometry

Cells with or without transfection were seeded into 60 mm culture dishes or 96-well plates and treated with Sch B or 5-FU for 48 h. The spent medium was collected, and the cells were harvested using trypsin-EDTA (0.05%). DMEM containing 10% FBS was used to inactivate the trypsin. The cells and spent medium were centrifuged at 1000 rpm for 3 min. The cell pellet was washed thrice with PBS and resuspended in 1× Annexin V binding buffer. The cell suspension was transferred to a 5 mL culture tube or 96-well plate, where PI stain and Annexin V were added according to the manufacturer’s instructions (Molecular Probes Dead Cell Apoptosis Kits with Annexin V for Flow Cytometry, Thermo Fisher Scientific). A BD Bioscience FacsAria III (San Jose, CA) or Beckman Coulter Cytoflex S flow cytometer (Brea, CA) was used to analyze the fluorescence output at the FITC and PI emission wavelengths. The fluorescence intensity of 10,000 cells was recorded. FlowJo 7.6 or CytoFLEX CytExpert 2.3 software was used for the data analysis.

### Colony Formation Assay

Cells (10^6^ cells/well) were seeded into 6-well plates and treated with Sch B or 5-FU for 48 h. At the end of treatment, the treated cells were trypsinized, and 500 cells were replated into clean 6-well plates and allowed to grow for an additional 14 days. The formed colonies were fixed with 100% ethanol and stained with 1% crystal violet (Sigma) before being captured and counted manually.

### BrdU-ELISA assay

Cells were seeded at a 10^4^ cells/well concentration onto 96-well culture plates and treated with Sch B for 48 h. The BrdU Cell Proliferation ELISA Kit (ab126556, Abcam) was used according to the manufacturer’s instructions. 10 μL of 10× BrdU solution was added to the cells, which were then incubated for 4 h. After washing with PBS, the cells were fixed with a fixing solution. Next, an anti-BrdU monoclonal detector antibody (100 μL/well) was added, and the plate was incubated for 1 h at room temperature. Finally, a spectrophotometric microtiter plate reader was used to measure the absorbance at 450 nm to identify cell proliferation. BrdU incorporation in each sample was calculated as the OD of the treatment sample minus the mean OD of the control without the addition of BrdU.

### Real-Time Quantitative Polymerase Chain Reaction (RT-qPCR)

HCT116 cells (6 × 10^5^ cells/well) were treated with various concentrations of Sch B for 48 h. The RNAiso Plus or GeneJET RNA Purification kit was used to extract total RNA, according to the manufacturer’s instructions. Purified RNA was resuspended in 50 μL nuclease-free water and stored at −80 °C. RNA concentrations were measured using a NanoDrop ND-1000 Spectrophotometer (Nano-Drop Technologies, Wilmington, DE). RNA purity was determined by ensuring a value of 1.8–2.0 for the A260/A280 ratio. Complementary DNA (cDNA) was prepared from 500 ng of total RNA using the HiScript RT SuperMix or PrimeScript RT reagent kit with gDNA Eraser for qPCR, according to the manufacturer’s instructions. qPCR was performed to quantify the mRNA expression levels of ER stress- (*DDIT3*, *ATF3*, *ATF5*, *SMAD3*, *HSPA5*) and apoptosis-related genes (*BAX*, *BCL2*, *CASP3*). Glyceraldehyde-3-phosphate dehydrogenase (GAPDH) levels were also assessed as an internal control for RNA integrity and loading. No differences in *GAPDH* levels were found between the groups for any of the genes investigated (data not shown). All samples were run on a StepOnePlus Real-Time PCR system (Applied Biosystems, Foster City, CA) or LightCycler 96 System (Roche, Burgess Hill, UK) using 2 μL of cDNA and AceQ qPCR SYBR Green Master Mix or TB Green Premix Ex Taq, with final primer concentrations of 0.5 μM per primer in a final volume of 10 μL. PCRs were performed using the default fast program (45 cycles of 95 °C for 5 s and 60 °C for 30 s). To ensure the reliability of RT-qPCR data, amplicons were kept short (<250 bp). Amplification specificity was checked with melting curve analysis and gel electrophoresis. All PCRs were performed in duplicates. Relative changes in the gene expression levels in cultured intestinal cells were analyzed using the 2^–△△CT^ method, as described previously.^38^ Human-specific primers are described in Table S2.

### Protein Extraction, SDS-PAGE, and Western Blotting

Cells were seeded into 60 mm culture dishes or 6-well plates and treated with Sch B for 48 h. The cells were lysed with RIPA lysis buffer, supplemented with a protease inhibitor cocktail, and total protein was extracted. Protein levels were normalized using the DC Protein Assay (BioRad). 20–50 μg proteins were loaded onto a 10% SDS-PAGE gel and blotted onto a PDVF membrane. The membrane was blocked with 5% dry milk (w/v) in Tris-buffered saline (TBS), containing 0.05% (v/v) Tween 20 (TBST) buffer for 1 h at room temperature. Proteins were probed with primary antibodies, anti-BAX (1:1000, sc-7480, Santa Cruz, Dallas, TX), anti-BCL-2 (1:1000, sc-7382, Santa Cruz, Beverly, MA), anti-caspase-3 (1:1000, 9662S, Cell Signaling Technology), and anti-cleaved caspase-3 (1:1000, 9664S, Cell Signaling Technology) overnight at 4 °C. The membrane was washed five times with TBST for 5 min each and incubated with anti-rabbit (1:1000, 1706515, BioRad) or anti-mouse (1:1000, 1721011, BioRad) secondary antibodies for 1 h at room temperature. The membrane was washed five times with TBST prior to visualization using the Clarity Western ECL Substrate kit (BioRad). Chemiluminescence was detected with a digital imaging system (ChemiDoc XRS+ system, Bio-Rad). Band intensity was quantified by ImageJ (NIH, Bethesda, MD). β-Actin (1:1000, 1 h at room temperature, #3700, Cell Signaling Technology) was used as a loading control.

### Raman Spectroscopy

The Sch B powder sample was first analyzed with a conventional benchtop Renishaw InVia Qontor Raman spectrometer (Renishaw, Wotton-under-Edge, Gloucestershire, UK) with an excitation wavelength of 785 nm and sample acquisition of 10 s. Cells were treated with or without Sch B and lysed in ice-cold DMEM, followed by passage through syringe needles at least three times. The samples were then analyzed by using an in-house compact Raman spectrometer with an excitation wavelength of 785 nm. Although the in-house system had a lower spectral resolution than the conventional one, the in-house Raman system provided flexibility and ease of measuring cellular suspensions directly with higher reproducibility. The measurements were performed using a quartz cuvette sample holder and a signal acquisition of 100 s.

### Computational Docking

Molecular docking of the Sch B molecule into the CHOP protein was carried out using the Molecular Operating Environment (MOE) program. The Sch B molecule was built using the “Builder” function in MOE, and the structure for the CHOP protein was obtained from the AlphaFold Protein Structure Database (AlphaFold DB) (Uniprot ref: P35638). Docking sites were identified using the “Site Finder” tool, which identifies potential sites based on an α sphere approach. Docking was performed using the “Dock” module, using the Triangle Matcher placement methodology and 100 placement poses, with optimization carried out using a molecular mechanics method using the Amber 10 force field. The binding energy (E_refine) was determined from the change in the nonbonded energy between the ligand and receptor upon the addition of the ligand. The binding free energy (E_score2) was determined using the GBVI/WSA scoring function.

### siRNA Transfection

Cells were transfected with siRNA using Lipofectamine RNAiMAX reagent (Thermo Fisher Scientific) according to the manufacturer’s instructions. The knockdown efficiency was validated by qRT-PCR and Western blot analyses. After 72 h of incubation, transfected cells were treated with or without Sch B for 48 h and harvested for RNA and protein extraction, CCK cell viability assay, and Annexin V/PI flow cytometry.

### Immunofluorescence

HCT116 cells were fixed (3.7% formaldehyde, 10 min), permeabilized (0.25% Triton X-100, 5% FBS, 15 min), blocked (5% FBS for 1 h at room temperature), and incubated with anti-DDIT3/GADD153/CHOP (1:200, sc-56107, Santa Cruz) in 5% FBS overnight at 4 °C, and Alexa Fluor 488 goat anti-mouse IgG secondary antibody (1:200, #10696113, Thermo Fisher Scientific) for 1 h at room temperature. Slides were counterstained and mounted (Fluoromount-G Mounting Medium, #5596276, Thermo Fisher Scientific), imaged by a Zeiss Axiovert LSM 710 VIS40S confocal microscope, and quantified by ImageJ software.

### Mouse Xenograft Model of Human Colon Cancer

Five-week-old BALB/c nude mice, weighing 18–20 g, were obtained from the Laboratory Animal Unit. The animals were housed in individually ventilated cages. The temperature was kept at 22 ± 2 °C, with a 12 h light/dark cycle. Animals were fed a standard diet (AIN-93G, Research Diets, New Brunswick, NJ) and given water *ad libitum*. After 1 week of acclimatization, HCT116 cells were injected subcutaneously into the mice. HCT116 cells were suspended in PBS at a concentration of 1 × 10^7^ cells/mL. 200 μL of the cell suspension was injected into the right flanks of the mice. One week was allowed for the tumor formation. Then, the mice were randomized into different groups, including PBS (Sham), Sch B (50 mg/kg), and 5-FU positive control (75 mg/kg). Sch B was administered by oral gavage every other day, while 5-FU was given by intraperitoneal injection once a week (start of the week). The treatment lasted for 14 days, and body weight, tumor volume, and feces were observed throughout the period. Tumor volumes were calculated based on the following formula: volume [mm^3^] = π × length × width^2^/6. Length represents the longest tumor diameter (mm), and width represents the perpendicular diameter (mm). Mice were sacrificed using an overdose of pentobarbital sodium (Dorminal, 250 mg/kg, intraperitoneal injection, Alfasan, Woerden, The Netherlands). Tumors were removed and snap-frozen in liquid nitrogen or fixed in 10% neutral-buffered formalin at 4 °C overnight. All study protocols were approved by the Committee on the Use of Live Animals in Teaching and Research (CULATR No. 5068-19) of the University of Hong Kong and the Department of Health of the HKSAR Government.

### Hematoxylin and Eosin and Immunohistochemistry Staining

Tumors were harvested at the end of sacrifice and fixed in 10% neutral-buffered formalin at 4 °C overnight. THe fixed tumor tissues were then embedded in paraffin wax using a Leica Tissue Processor (model ASP300S, Leica Microsystems Inc., Wetzlar, Germany). 5 μm sections were deparaffinized and rehydrated in xylene, 1:1 xylene:ethanol, graded ethanol (100% twice, 95%, 70%, and 50%), and distilled water for 5 min each. Hematoxylin and eosin (H&E) staining was performed by using the Hematoxylin–Eosin Stain kit (H-3502, Vector Laboratories, CA) according to the manufacturer’s instructions. The stained slides were mounted with Histomount.

For immunohistochemistry, 5 μm sections were deparaffinized and rehydrated, as described above. Antigen retrieval was done by heating slides in 10 mM sodium citrate buffer (pH 6.0) at 95 °C for 20 min. Slides were then blocked in 1% BSA for 2 h and 3% hydrogen peroxide for 15 min to reduce nonspecific binding and inactivate endogenous peroxidase activities followed by incubation with anti-Ki67 primary antibody (1:5000, ab15580, Abcam) overnight at 4 °C. The sections were then washed and incubated with biotinylated anti-rabbit secondary antibody (1:1000, ab64256, Abcam) for 1 h at room temperature. The sections were washed with PBST and then incubated with streptavidin–horseradish peroxidase (HRP), visualized with DAB substrate (DAB Substrate Kit, ab64238, Abcam), counterstained with hematoxylin (Vector Laboratories), and mounted with Histomount. Images were captured using a Nikon 80i fluorescence microscope (Nikon USA, Melville, NY) at 200× magnification. Intensity of Ki-67 staining was quantified by using ImageJ software as described previously.^39^

### mRNA Sequencing and Data Analysis

Total RNA was extracted using a Qiagen RNeasy Kit (#74004, Qiagen, Hilden, Germany). Quantity and quality were determined by an Agilent 2100 bioanalyzer (G2939A, Agilent Technologies, Waldbronn, Germany) before RNA-seq library preparation. Paired-end sequencing with 100 bases read length was performed using the BGI DNBSEQ platform (Wuhan, China). Low-quality reads (more than 20% of the bases qualities were lower than 10), reads with adaptors, and reads with unknown bases (N bases more than 5%) were filtered using SOAPnuke (v1.5.2) (parameters: -l 20 -q 0.4 -n 0.1 -Q 2 -5 1). HISAT2 (v2.0.4) was used for genome mapping (parameters: --phred33 --sensitive --no-discordant --no-mixed -I 1 -X 1000). Clean reads were mapped onto the reference genome (hg19). StringTie (v1.0.4) was used to reconstruct transcripts, and Cuffcompare (Cufflink v2.2.1 tools) was used to compare the reconstructed transcripts to reference annotations. CPC (v0.9-r2) was used to predict the coding potential of novel transcripts. The clean reads were mapped to reference using Bowtie2 (v2.2.5), and the gene expression levels of each sample were calculated with RSEM (v1.2.12) to conduct alignments and calculate the FPKM value of genes, respectively.^40^ Differentially expressed genes were identified with DEseq2, and genes with an absolute fold change of log_2_-transformed values ≥1.5, and a *P*-value threshold <0.05 were deemed as differentially expressed genes. Functional changes and activated canonical pathways were analyzed with ingenuity pathways analysis (IPA, Qiagen).

### Statistical Analysis

GraphPad Prism 9 was used for statistical analysis (GraphPad, Boston, MA). Normality tests were performed to determine whether parametric or nonparametric tests were used for the analyses. For parametric data, either two-tailed Student’s *t-*test, one-way, or two-way ANOVA with Holm–Šídák’s multiple comparison test was used. For nonparametric data, either the two-tailed Mann–Whitney *U*-test or Kruskal–Wallis test with Dunn’s correction was used, and paired data were analyzed by the Wilcoxon-matched-pair signed-rank test. Data were displayed as mean ± SD. * indicated a *P*-value of less than 0.05, ** indicated *P* < 0.01, and *** indicated *P* < 0.001. The quantification of confocal microscopy data was based on the mean fluorescence intensity of 50 cells from independent experiments.

## Data Availability

The data that support the findings of this study are available from the corresponding author upon reasonable request.

## Supporting Information Available

Effect of Schisandrin B on the viability of human colon cancer cell lines and the normal human intestinal cell line by the Cell Counting Kit-8 assay; effect of Schisandrin B on Ki-67 protein expression in tumor tissues obtained from mice treated with Schisandrin B or 5-fluorouracil compared to PBS sham by immunohistochemistry; biofunction analysis identified a number of genes responsible for the inhibition of cell proliferation and activation of apoptosis in tumor tissues obtained from Schisandrin B-treated mice; top canonical pathways and lack of activation of unfolded protein response pathway by Schisandrin B in tumors of nude mice; binding energies for Schisandrin B at the nine docking sites identified in the CHOP protein using the site finder tool in MOE; qRT-PCR primers used in this study. (PDF)

## References

[ref1] Sung H.; Ferlay J.; Siegel R.; Laversanne M.; Soerjomataram I.; Jemal A.; Bray F. Global cancer statistics 2020: GLOBOCAN estimates of incidence and mortality worldwide for 36 cancers in 185 countries. CA: Cancer J. Clin. 2021, 71 (3), 209–249. 10.3322/caac.21660. 33538338

[ref2] Morgan E.; Arnold M.; Gini A.; Lorenzoni V.; Cabasag C.; Laversanne M.; Vignat J.; Ferlay J.; Murphy N.; Bray F. Global burden of colorectal cancer in 2020 and 2040: incidence and mortality estimates from GLOBOCAN. Gut 2023, 72 (2), 338–344. 10.1136/gutjnl-2022-327736. 36604116

[ref3] Monson J.; Weiser M.; Buie W.; Chang G.; Rafferty J.; Buie W.; Rafferty J.; et al. Practice parameters for the management of rectal cancer (revised). Dis. Colon Rectum 2013, 56 (5), 535–550. 10.1097/DCR.0b013e31828cb66c. 23575392

[ref4] Wu Y.; Deng Z.; Wang H.; Ma W.; Zhou C.; Zhang S. Repeated cycles of 5-fluorouracil chemotherapy impaired anti-tumor functions of cytotoxic T cells in a CT26 tumor-bearing mouse model. BMC Immunol. 2016, 17 (1), 29 10.1186/s12865-016-0167-7. 27645787 PMC5028929

[ref5] Patra S.; Pradhan B.; Nayak R.; Behera C.; Das S.; Patra S.; Efferth T.; Jena M.; Bhutia S. Dietary polyphenols in chemoprevention and synergistic effect in cancer: clinical evidences and molecular mechanisms of action. Phytomedicine 2021, 90, 153554 10.1016/j.phymed.2021.153554. 34371479

[ref6] Zhou Y.; Zheng J.; Li Y.; Xu D.; Li S.; Chen Y.; Li H. Natural polyphenols for prevention and treatment of cancer. Nutrients 2016, 8 (8), 515 10.3390/nu8080515. 27556486 PMC4997428

[ref7] Rudrapal M.; Khairnar S.; Khan J.; Dukhyil A.; Ansari M.; Alomary M.; Alshabrmi F.; Palai S.; Deb P.; Devi R. Dietary polyphenols and their role in oxidative stress-induced human diseases: insights into protective effects, antioxidant potentials and mechanism (s) of action. Front. Pharmacol. 2022, 13, 806470 10.3389/fphar.2022.806470. 35237163 PMC8882865

[ref8] León-González A. J.; Auger C.; Schini-Kerth V. Pro-oxidant activity of polyphenols and its implication on cancer chemoprevention and chemotherapy. Biochem. Pharmacol. 2015, 98 (3), 371–380. 10.1016/j.bcp.2015.07.017. 26206193

[ref9] Long J.; Guan P.; Hu X.; Yang L.; He L.; Lin Q.; Luo F.; Li J.; He X.; Du Z.; Li T. Natural polyphenols as targeted modulators in colon cancer: molecular mechanisms and applications. Front. Immunol. 2021, 12, 635484 10.3389/fimmu.2021.635484. 33664749 PMC7921316

[ref10] Núñez-Sánchez M. A.; González-Sarrías A.; Romo-Vaquero M.; García-Villalba R.; Selma M.; Tomás-Barberán F.; García-Conesa M.; Espín J. Dietary phenolics against colorectal cancer—From promising preclinical results to poor translation into clinical trials: Pitfalls and future needs. Mol. Nutr Food Res. 2015, 59 (7), 1274–1291. 10.1002/mnfr.201400866. 25693744

[ref11] Bracci L.; Fabbri A.; Del Cornò M.; Conti L. Dietary polyphenols: promising adjuvants for colorectal cancer therapies. Cancers 2021, 13 (18), 4499 10.3390/cancers13184499. 34572726 PMC8465098

[ref12] Yang X.; Wang S.; Mu Y.; Zheng Y. Schisandrin B inhibits cell proliferation and induces apoptosis in human cholangiocarcinoma cells. Oncol. Rep. 2016, 36 (4), 1799–1806. 10.3892/or.2016.4992. 27499090 PMC5022873

[ref13] Liu Z.; Zhang B.; Liu K.; Ding Z.; Hu X. Schisandrin B attenuates cancer invasion and metastasis via inhibiting epithelial-mesenchymal transition. PLoS One 2012, 7 (7), e40480 10.1371/journal.pone.0040480. 22848381 PMC3405072

[ref14] Dai X.; Yin C.; Guo G.; Zhang Y.; Zhao C.; Qian J.; Wang O.; Zhang X.; Liang G. Schisandrin B exhibits potent anticancer activity in triple negative breast cancer by inhibiting STAT3. Toxicol. Appl. Pharmacol. 2018, 358, 110–119. 10.1016/j.taap.2018.09.005. 30195018

[ref15] Wang S.; Wang A.; Shao M.; Lin L.; Li P.; Wang Y. Schisandrin B reverses doxorubicin resistance through inhibiting P-glycoprotein and promoting proteasome-mediated degradation of survivin. Sci. Rep. 2017, 7 (1), 8419 10.1038/s41598-017-08817-x. 28827665 PMC5567212

[ref16] Li Q.; Lu X.; Wang C.; Cai L.; Lu J.; Wu J.; Zhuge Q.; Zheng W.; Su Z. Antiproliferative and apoptosis-inducing activity of schisandrin B against human glioma cells. Cancer Cell Int. 2015, 15, 12 10.1186/s12935-015-0160-x. 25685066 PMC4326453

[ref17] Wang B.; Wang X.; Tong X.; Zhang Y. Schisandrin B inhibits cell viability and migration, and induces cell apoptosis by circ_0009112/miR-708–5p axis through PI3K/AKT pathway in osteosarcoma. Front. Genet. 2020, 11, 588670 10.3389/fgene.2020.588670. 33414806 PMC7783358

[ref18] Liu X. N.; Zhang C. Y.; Jin X. D.; Li Y. Z.; Zheng X. Z.; Li L. Inhibitory effect of schisandrin B on gastric cancer cells in vitro. World J. Gastroenterol. 2007, 13 (48), 6506–6511. 10.3748/wjg.v13.i48.6506. 18161920 PMC4611289

[ref19] He L.; Chen H.; Qi Q.; Wu N.; Wang Y.; Chen M.; Feng Q.; Dong B.; Jin R.; Jiang L. Schisandrin B suppresses gastric cancer cell growth and enhances the efficacy of chemotherapy drug 5-FU in vitro and in vivo. Eur. J. Pharmacol. 2022, 920, 174823 10.1016/j.ejphar.2022.174823. 35157912

[ref20] Pu Z.; Zhang W.; Wang M.; Xu M.; Xie H.; Zhao J. Schisandrin B attenuates colitis-associated colorectal cancer through SIRT1 linked SMURF2 signaling. Am. J. Chin. Med. 2021, 49 (7), 1773–1789. 10.1142/S0192415X21500841. 34632965

[ref21] Li J.; Lu Y.; Wang D.; Quan F.; Chen X.; Sun R.; Zhao S.; Yang Z.; Tao W.; Ding D.; et al. Schisandrin B prevents ulcerative colitis and colitis-associated-cancer by activating focal adhesion kinase and influence on gut microbiota in an in vivo and in vitro model. Eur. J. Pharmacol. 2019, 854, 9–21. 10.1016/j.ejphar.2019.03.059. 30951716

[ref22] Xiang S. S.; Wang X. A.; Li H. F.; Shu Y. J.; Bao R. F.; Zhang F.; Cao Y.; Ye Y. Y.; Weng H.; Wu W. G.; et al. Schisandrin B induces apoptosis and cell cycle arrest of gallbladder cancer cells. Molecules 2014, 19 (9), 13235–13250. 10.3390/molecules190913235. 25165862 PMC6271519

[ref23] Wang Y.; Chen J.; Huang Y.; Yang S.; Tan T.; Wang N.; Zhang J.; Ye C.; Wei M.; Luo J.; Luo X. Schisandrin B suppresses osteosarcoma lung metastasis in vivo by inhibiting the activation of the Wnt/β-catenin and PI3K/Akt signaling pathways. Oncol. Rep. 2022, 47 (3), 50 10.3892/or.2022.8261. 35029287 PMC8771162

[ref24] Li S.; Wang H.; Ma R.; Wang L. Schisandrin B inhibits epithelial-mesenchymal transition and stemness of large-cell lung cancer cells and tumorigenesis in xenografts via inhibiting the NF-κB and p38 MAPK signaling pathways. Oncol. Rep. 2021, 45 (6), 115 10.3892/or.2021.8066. 33907830 PMC8107653

[ref25] Molecular Operating Environment (MOE); Chemical Computing Group Inc.: Montreal, QC, Canada, 2016.

[ref26] Zeeshan H. M.; Lee G. H.; Kim H. R.; Chae H. J. Endoplasmic reticulum stress and associated ROS. Int. J. Mol. Sci. 2016, 17 (3), 327 10.3390/ijms17030327. 26950115 PMC4813189

[ref27] Dijkstra E. A.; Hospers G.; Kranenbarg E.; Fleer J.; Roodvoets A.; Bahadoer R.; Guren M.; Tjalma J.; Putter H.; Crolla R.; et al. Quality of life and late toxicity after short-course radiotherapy followed by chemotherapy or chemoradiotherapy for locally advanced rectal cancer–The RAPIDO trial. Radiother. Oncol. 2022, 171, 69–76. 10.1016/j.radonc.2022.04.013. 35447283

[ref28] Maertens O.; McCurrach M.; Braun B.; De Raedt T.; Epstein I.; Huang T.; Lauchle J.; Lee H.; Wu J.; Cripe T.; et al. A collaborative model for accelerating the discovery and translation of cancer therapies. Cancer Res. 2017, 77 (21), 5706–5711. 10.1158/0008-5472.CAN-17-1789. 28993414 PMC5668167

[ref29] Suh D.-C.; Powers C.; Barone J.; Shin H.; Kwon J.; Goodin S. Full costs of dispensing and administering fluorouracil chemotherapy for outpatients: A microcosting study. Res. Social Adm. Pharm. 2010, 6 (3), 246–256. 10.1016/j.sapharm.2009.07.004. 20813337 PMC5292979

[ref30] Oakes S. A. Endoplasmic Reticulum Stress Signaling in Cancer Cells. Am. J. Pathol. 2020, 190 (5), 934–946. 10.1016/j.ajpath.2020.01.010. 32112719 PMC7237829

[ref31] Yadav R. K.; Chae S. W.; Kim H. R.; Chae H. J. Endoplasmic reticulum stress and cancer. J. Cancer Prev. 2014, 19 (2), 75–88. 10.15430/JCP.2014.19.2.75. 25337575 PMC4204165

[ref32] Jackson K. G.; Way G.; Zeng J.; Lipp M.; Zhou H. The dynamic role of endoplasmic reticulum stress in chronic liver disease. Am. J. Pathol. 2023, 193, 1389–1399. 10.1016/j.ajpath.2023.03.009. 37028592 PMC10548273

[ref33] Rozpedek W.; Pytel D.; Mucha B.; Leszczynska H.; Diehl J. A.; Majsterek I. The role of the PERK/eIF2α/ATF4/CHOP signaling pathway in tumor progression during endoplasmic reticulum stress. Curr. Mol. Med. 2016, 16 (6), 533–544. 10.2174/1566524016666160523143937. 27211800 PMC5008685

[ref34] Hu H.; Tian M.; Ding C.; Yu S. The C/EBP homologous protein (CHOP) transcription factor functions in endoplasmic reticulum stress-induced apoptosis and microbial infection. Front. Immunol. 2019, 9, 3083 10.3389/fimmu.2018.03083. 30662442 PMC6328441

[ref35] Bromati C. R.; Lellis-Santos C.; Yamanaka T.; Nogueira T.; Leonelli M.; Caperuto L.; Gorjão R.; Leite A.; Anhê G.; Bordin S. UPR induces transient burst of apoptosis in islets of early lactating rats through reduced AKT phosphorylation via ATF4/CHOP stimulation of TRB3 expression. Am. J. Physiol Regul. Integr. Comp. Physiol. 2011, 300 (1), R92–R100. 10.1152/ajpregu.00169.2010. 21068199

[ref36] Gotoh T.; Terada K.; Oyadomari S.; Mori M. hsp70-DnaJ chaperone pair prevents nitric oxide- and CHOP-induced apoptosis by inhibiting translocation of Bax to mitochondria. Cell Death Differ. 2004, 11 (4), 390–402. 10.1038/sj.cdd.4401369. 14752510

[ref37] Lee B.-R.; Chang S.; Hong E.; Kwon B.; Kim H.; Kim Y.; Lee J.; Cho H.; Cheon J.; Ko H. Elevated endoplasmic reticulum stress reinforced immunosuppression in the tumor microenvironment via myeloid-derived suppressor cells. Oncotarget 2014, 5 (23), 12331–12345. 10.18632/oncotarget.2589. 25514597 PMC4322987

[ref38] Livak K. J.; Schmittgen T. D. Analysis of relative gene expression data using real-time quantitative PCR and the 2- ΔΔCT method. Methods 2001, 25 (4), 402–408. 10.1006/meth.2001.1262. 11846609

[ref39] Crowe A. R.; Yue W. Semi-quantitative determination of protein expression using immunohistochemistry staining and analysis: an integrated protocol. Bio Protoc. 2019, 9 (24), e3465 10.21769/BioProtoc.3465. PMC6924920 31867411

[ref40] Love M. I.; Huber W.; Anders S. Moderated estimation of fold change and dispersion for RNA-seq data with DESeq2. Genome Biol. 2014, 15 (12), 550 10.1186/s13059-014-0550-8. 25516281 PMC4302049

