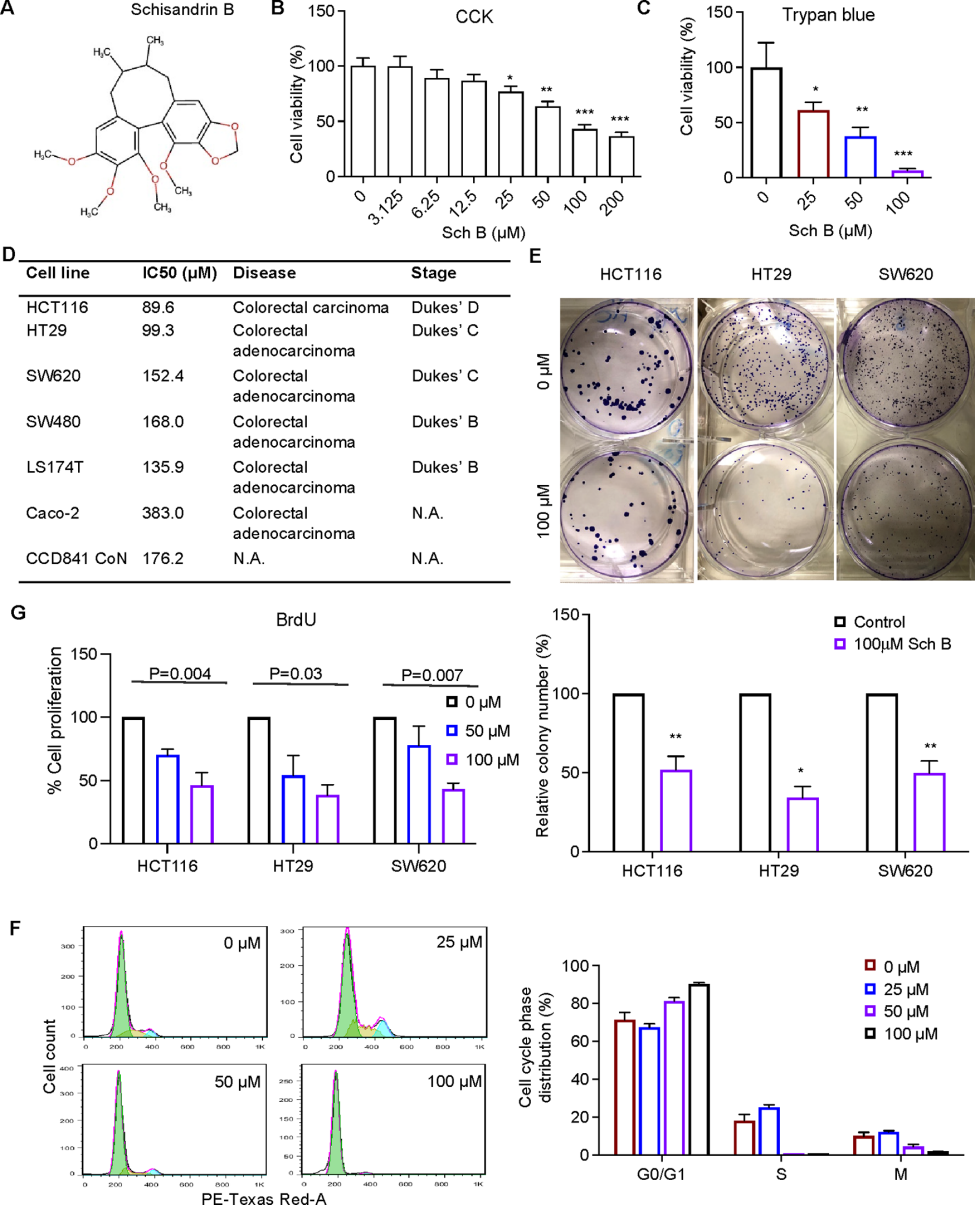# Correction to “Schisandrin B Suppresses Colon Cancer Growth by Inducing Cell Cycle Arrest and Apoptosis: Molecular Mechanism and Therapeutic Potential”

**DOI:** 10.1021/acsptsci.4c00580

**Published:** 2024-10-28

**Authors:** Vanessa Anna Co, Hani El-Nezami, Yawen Liu, Bonsra Twum, Priyanka Dey, Paul A. Cox, Shalu Joseph, Roland Agbodjan-Dossou, Mehdi Sabzichi, Roger Draheim, Murphy Lam Yim Wan

Correction made to Figure 1 due to the incorrect chemical structure of Schisandrin B. The revised figure with correct chemical structure is shown below.