# Supplementary material for: Schisandrin B Suppresses Colon Cancer Growth by Inducing Cell Cycle Arrest and Apoptosis: Molecular Mechanism and Therapeutic Potential
Source: ACS Pharmacol Transl Sci. 2024 Feb 22;7(3):863–77. doi: 10.1021/acsptsci.4c00009 (PMC10928902; doi:10.1021/acsptsci.4c00009)
Supplement: Supplementary file 1 — pt4c00009_si_001.pdf [file pt4c00009_si_001.pdf]

Supplementary Information

Schisandrin B suppresses colon cancer growth by inducing cell cycle arrest and apoptosis: molecular mechanism and therapeutic potential

Vanessa Anna Co[1], Hani El-Nezami[1,2]*, Yawen Liu[3], Bonsra Twum[3], Priyanka Dey[3], Paul A Cox[3], Shalu Joseph[3], Roland Agbodjan-Dossou[3], Mehdi Sabzichi[3], Roger Draheim[3], Murphy Lam Yim Wan[3,4]*

[1]School of Biological Sciences, Faculty of Science, Kadoorie Biological Sciences Building, The University of Hong Kong, Pokfulam, Hong Kong

[2]Institute of Public Health and Clinical Nutrition, University of Eastern Finland, Kuopio, Finland

[3]School of Pharmacy and Biomedical Sciences, Faculty of Science and Health, University of Portsmouth, Portsmouth, United Kingdom

[4]Division of Microbiology, Immunology and Glycobiology, Department of Laboratory Medicine, Faculty of Medicine, Lund University, Lund, Sweden

*Correspondences

Dr Hani El-Nezami

School of Biological Sciences, Faculty of Science, Kadoorie Biological Sciences Building, The University of Hong Kong, Pokfulam, Hong Kong

Email: elnezami@hku.hk

Dr Murphy Lam Yim Wan

School of Pharmacy and Biomedical Sciences, Faculty of Science and Health, University of Portsmouth, Portsmouth, United Kingdom

Email: murphy.wan@port.ac.uk

Figure S1

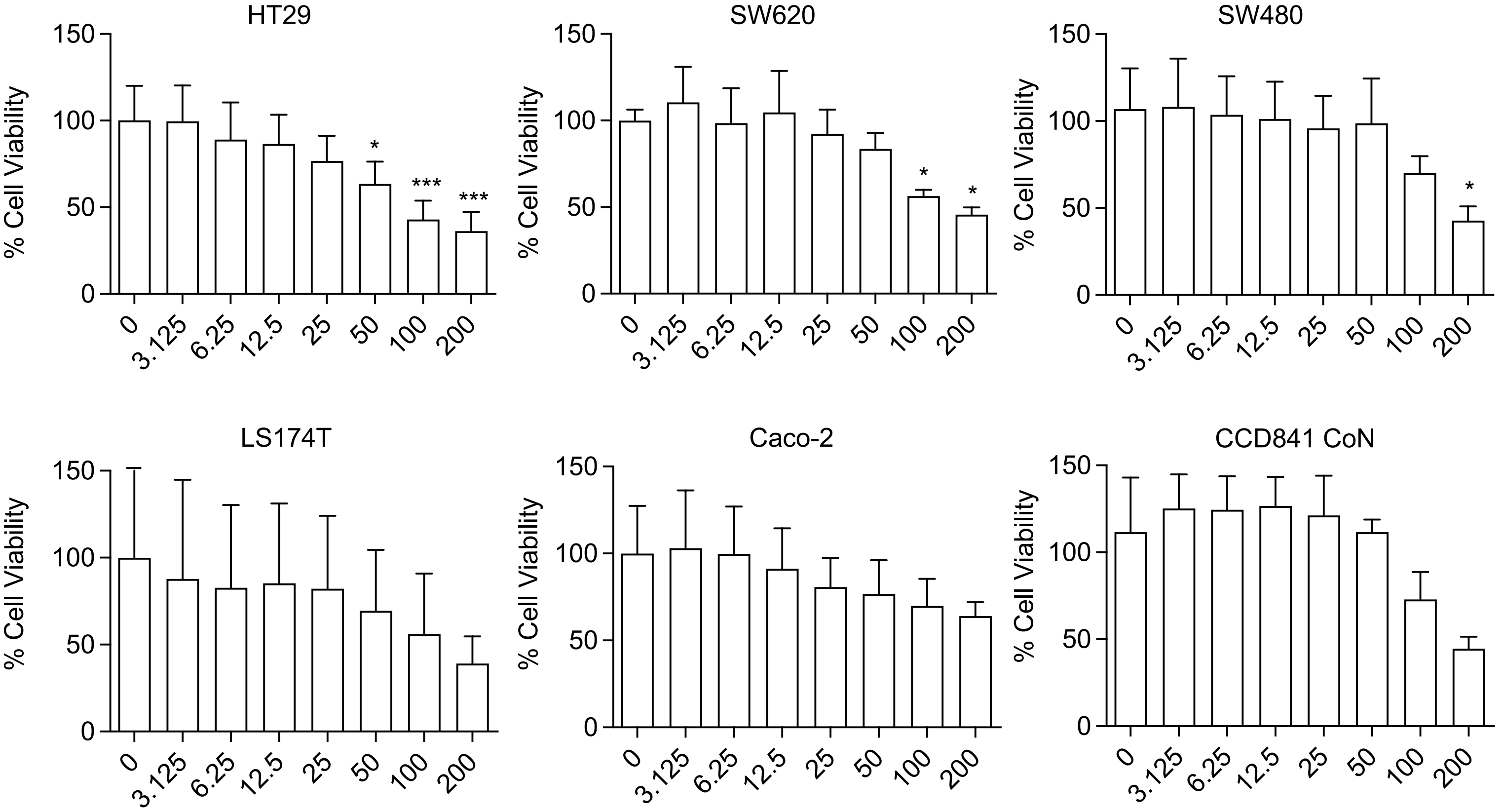

Figure S1. Supplementary data for Figure 1.

Effect of Schisandrin B (Sch B) on viability of human colon cancer cell lines (HT29, SW620, SW480, LS174T, Caco-2) and a normal human intestinal cell line (CCD841 CoN), quantified by the Cell Counting Kit-8 (CCK-8) assay (*n* = 3 experiments). Values were presented as mean ± SD, analyzed by Kruskal-Wallis test with Dunn’s correction. **P* < 0.05, ****P* < 0.001, compared with the control (i.e., 0 μM Sch B).

Figure S2

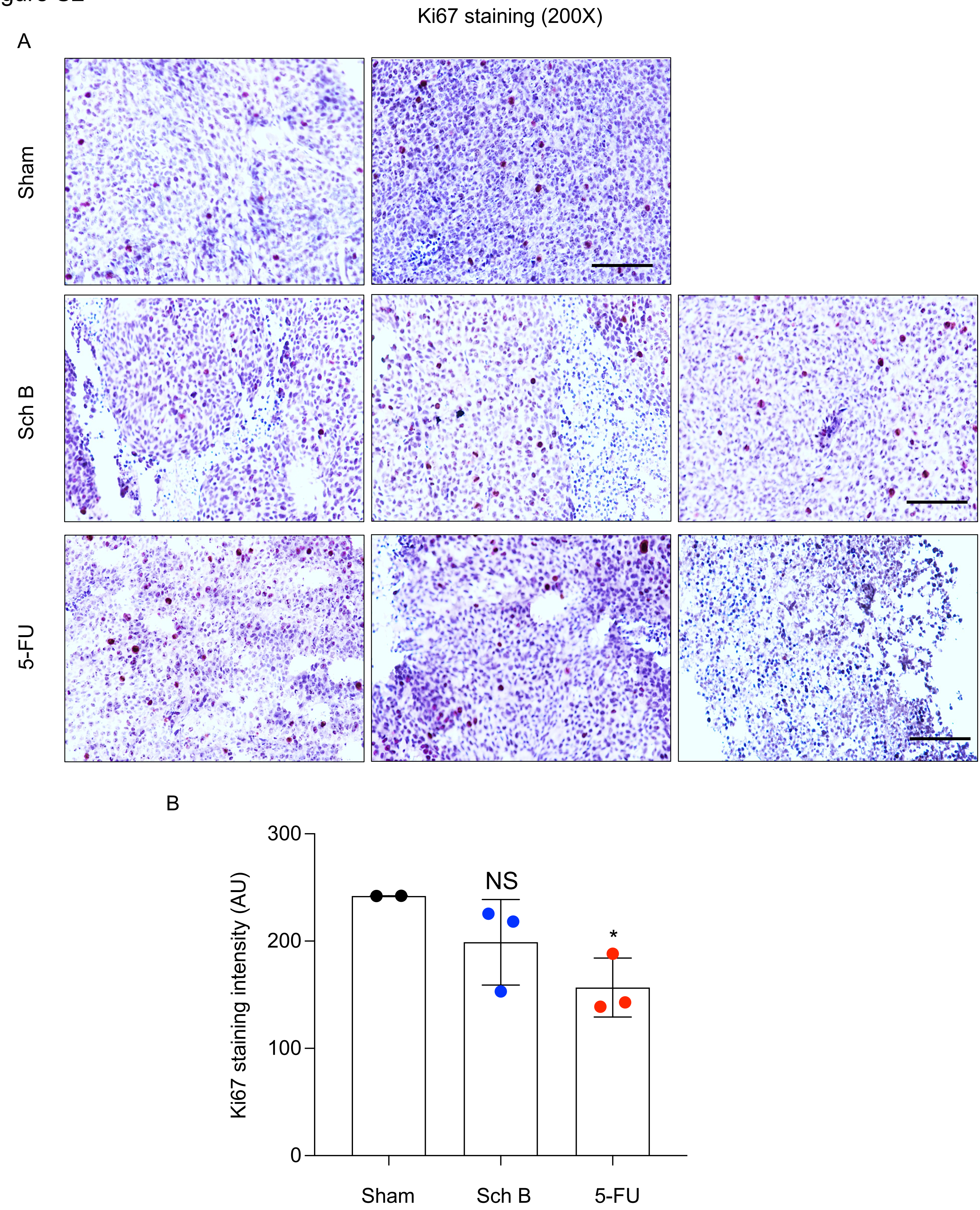

Figure S2. Supplementary data for Figure 7.

Effect of Schisandrin B (Sch B) on Ki-67 protein expression in tumor tissues obtained from mice treated with Sch B (50 mg/kg) or 5-FU (75 mg/kg) compared to PBS sham, quantified by the immunohistochemistry. (A) Representative sections (200x), scale bar 100 μm. (B) Quantification of Ki-67 staining from (A) ($n = 2$ - 3 mice per group). Values were presented as mean ± SD, analyzed by Kruskal-Wallis test with Dunn's correction. *$P < 0.05$, compared with the sham group. NS = Not significant.

Figure S3

Predicted inhibition of proliferation by Sch B treatment, $\log_2 FC > 1.5$, $P < 0.05$

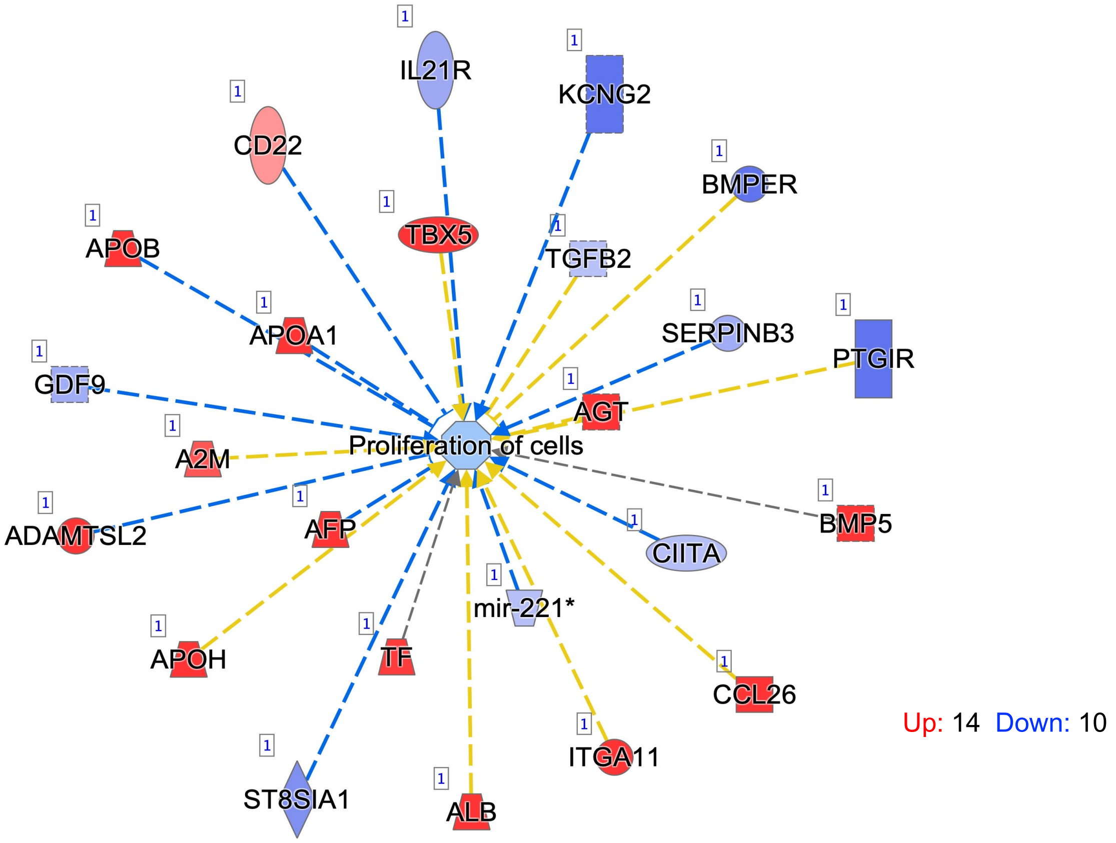

Predicted activation of apoptosis by Sch B treatment, $\log_2 FC > 1.5$, $P < 0.05$

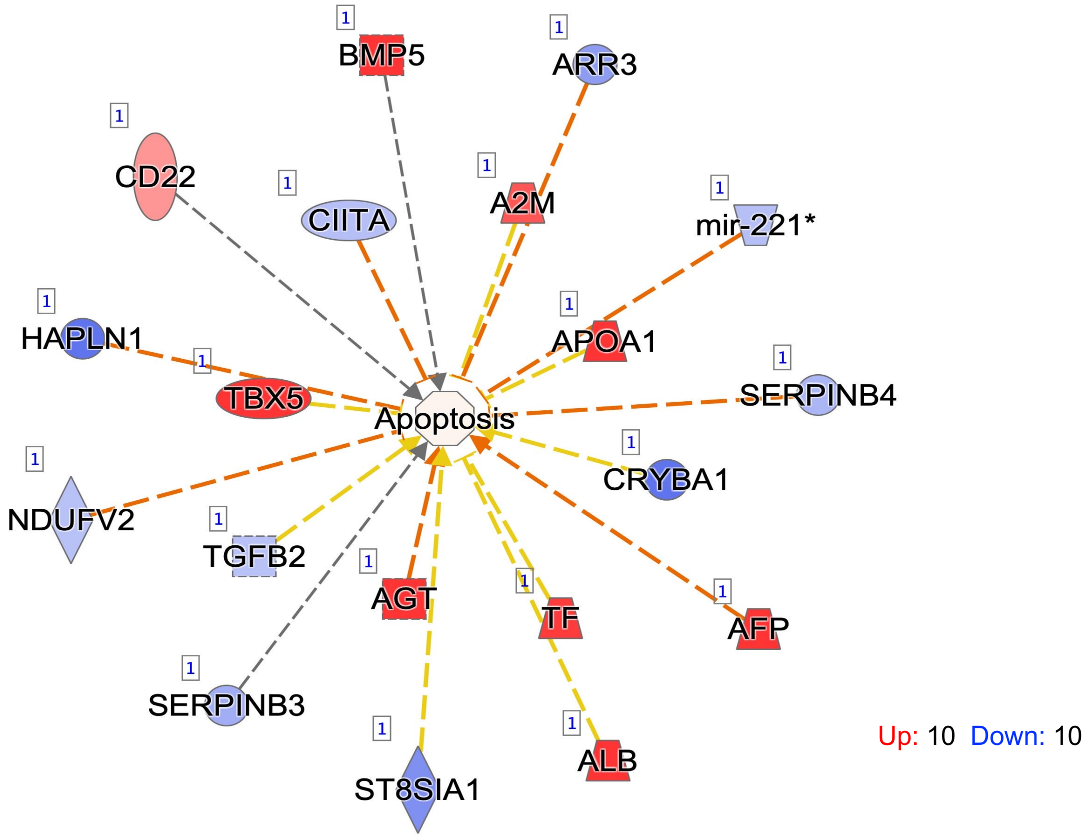

Figure S3. Supplementary data for Figure 7.

Biofunction analysis identified a number of genes responsible for the inhibition cell proliferation and activation of apoptosis in tumor tissues obtained from Sch B-treated mice. Cutoff $\log_2 FC \geq 1.5$, $P < 0.05$, $n = 3$ mice per group. Orange/yellow, activation of pathways; blue, inhibition of pathways; red, up-regulated genes; blue, down-regulated genes.

Figure S4

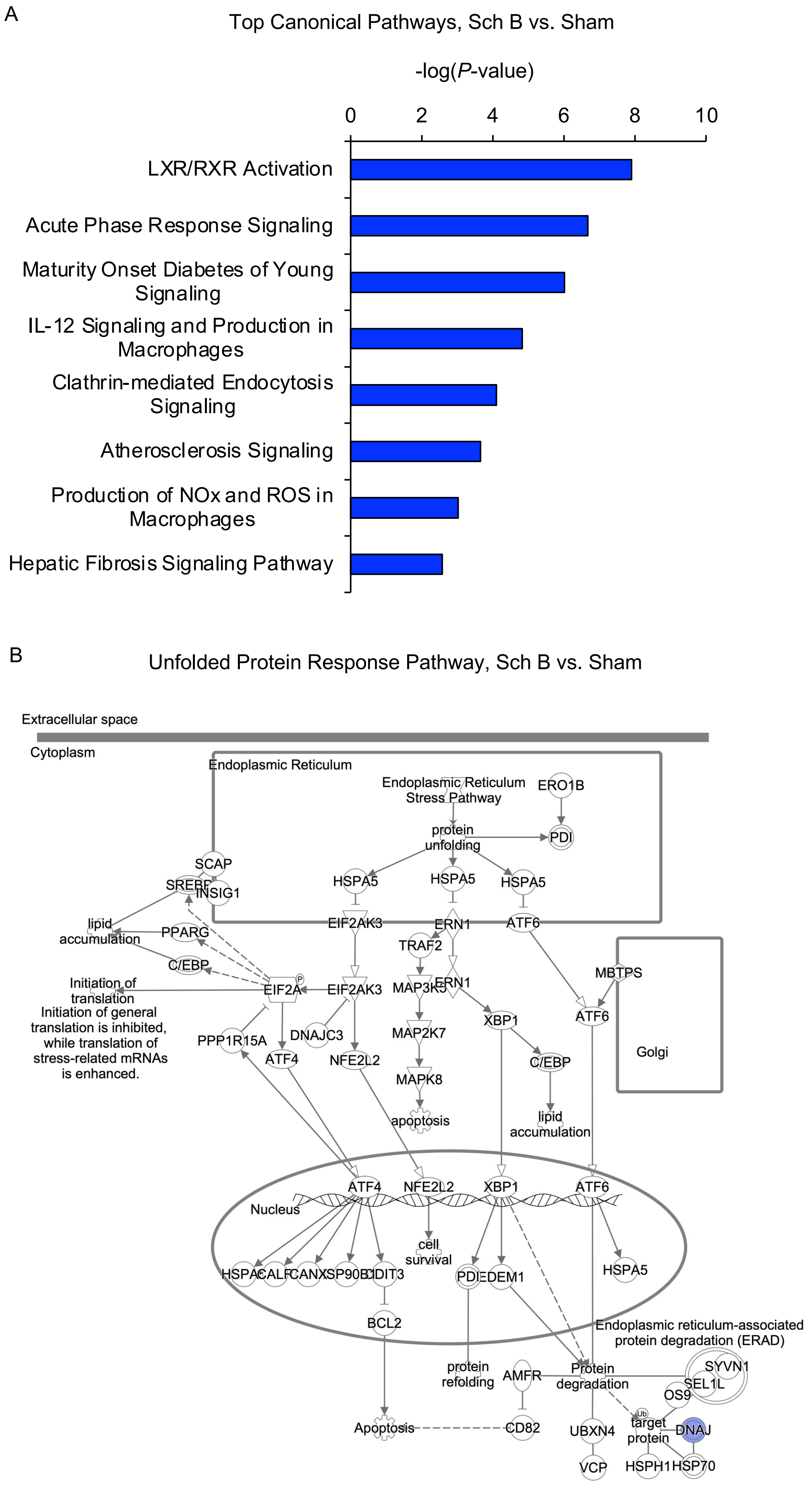

Figure S4. Supplementary data for Figure 6. (A) Top canonical pathways affected by Schisandrin B (Sch B) in tumors of nude mice. (B) Ingenuity pathway analysis showing lack of activation of unfolded protein response pathway in tumors of Sch B treated nude mice. Orange, up-regulated genes; blue, down-regulated genes.

Table S1. Binding energies for Sch B at the nine docking sites identified in the CHOP protein using the site finder tool in MOE

Site Number	Binding Energy kcal/mol	Binding Free Energy kcal/mol	Residues
1	-22.90	-5.07	PRO7 PHE8 SER9 PHE10 GLY11 THR12 LEU13
2	-23.63	-4.97	ARG140 GLN143 GLU144 GLU146 ARG147 ARG150
3	-28.54	-5.39	ASN44 GLU45 GLU46 GLU47 GLU48 SER49
4	-22.97	-5.01	MET120 LYS121 LYS123 GLU124 ASN127
5	-20.43	-4.73	VAL28 LEU29 ASP32 GLU33
6	-20.71	-4.71	GLN133 GLU136 GLU137 ARG140
7	-27.49	-5.31	GLU46 GLU47 SER49 LYS50
8	-21.55	-4.74	ALA115 GLN118 ARG119
9	-20.80	-4.80	LEU13 GLU17 LEU18 TRP21

Table S2. qRT-PCR primers used in this study

**Gene**	**Primer sequences**	**Reference/Accession Number**
BAX	Fwd 5'-CCTGTGCACCAAGGTGCCGGAACT-3'<br>Rev 5'-CCACCCTGGTCTTGGATCCAGCCC-3'	Elmaksoud *et al.* 2021, Biomedicine & Pharmacotherapy
BCL2	Fwd 5'-ATCGCCCTGTGGATGACTGAGT-3'<br>Rev 5'-GCCAGGAGAAATCAAACAGAGGC-3'	NM_000633
CASP3	Fwd 5'-GGAAGCGAATCAATGGACTCTGG-3'<br>Rev 5'-GCATCGACATCTGTACCAGACC-3'	NM_004346
DDIT3	Fwd 5'-GGTATGAGGACCTGCAAGAGGT-3'<br>Rev 5'-CTTGTGACCTCTGCTGGTTCTG-3'	NM_004083
SMAD3	Fwd 5'-TGAGGCTGTCTACCAGTTGACC-3'<br>Rev 5'-GTGAGGACCTTGTCAAGCCACT-3'	NM_005902
ATF3	Fwd 5'-CGCTGGAATCAGTCACTGTCAG-3'<br>Rev 5'-CTTGTTTCGGCACTTTGCAGCTG-3'	NM_001674
ATF5	Fwd 5'-GCTCGTAGACTATGGGAAACTCC-3'<br>Rev 5'-CATCCAGTCAGAGAAGCCATCAC-3'	NM_012068
HSPA5	Fwd 5'-CTGTCCAGGCTGGTGTGCTCT-3'<br>Rev 5'-CTTGGTAGGCACCACTGTGTTC-3'	NM_005347
GAPDH	Fwd 5'-ACCAGCCCCAGCAAGAGCACAAG-3'<br>Rev 5'-TTCAAGGGGTCTACATGGCAACTG-3'	Wang *et al.* 2006, Journal of Immunology
